# How does exposure to COVID-19 influence health and income inequality aversion?

**DOI:** 10.1007/s00355-023-01460-8

**Published:** 2023-05-19

**Authors:** Miqdad Asaria, Joan Costa-Font, Frank Cowell

**Affiliations:** 1 Department of Health Policy, London School of Economics and Political Science (LSE), London, UK; 2 Department of Economics and STICERD, London School of Economics and Political Science (LSE), London, UK; 3 IZA, Bonn, Germany; 4 CESIfo, Munich, Germany

## Abstract

We study individual aversion to health and income inequality in three European countries (the United Kingdom, Germany, and Italy), its determinants and especially, the effects of exposure to three types of COVID-19 specific shocks affecting individuals’ employment status, their income and health. Next, using evidence of representative samples of the population in the UK, we compare levels of health- and income-inequality aversion in the UK between the years 2016 and 2020. We document evidence of a significant increase in inequality aversion in both income and health domains. However, we show that inequality aversion is higher in the income domain than in the health domain. Furthermore, we find that inequality aversion in both domains increases in age and education and decreases in income and risk appetite. However, people directly exposed to major health shocks during the COVID-19 pandemic generally exhibited lower levels of aversion to both income and health inequality. Finally, we show that inequality aversion was significantly higher among those exposed to higher risk of COVID-19 mortality who experienced major health shocks during the pandemic.

**Supplementary Information:**

The online version contains supplementary material available at 10.1007/s00355-023-01460-8.

## Introduction

Inequality preferences play an important role in modern society as countries with higher inequality aversion might prioritise interventions reducing inequality. Inequality aversion reflects an individual concern with respect to the distribution of welfare in society, and may capture society’s evaluation of welfare loss from higher inequality (Atkinson 1970). By extension, one person’s degree of inequality aversion represents that person’s judgement about how far society should forgo increases in total outcomes to achieve a more egalitarian distribution.^1^ Inequality aversion can be thought of as a behavioural trait of groups such as countries or societies,^2^ rather than an individual specific parameter (Clark and D’Ambrosio 2014). Its role in informing policy is especially important when society experiences a significant collective health shock; so, it is important to understand the nature of inequality preferences in the context of a global pandemic.

Given that changes in people’s needs and circumstances arising from exogenous shocks may modify their tolerance of inequality, this paper contributes to the study of inequality aversion, focusing on the effects of the COVID-19 pandemic. However, we attempt to unpick the causal mechanism determining attitudes towards inequalities in income and life expectancy. Evidence from comparable shocks such as wartime exposure, reveal that war has a deep effect on social preferences and within-group cooperation (Gneezy and Fessler 2012).

Inequality preferences should not be assumed to be uniform across different domains of human experience. The “specific egalitarianism hypothesis” suggests that the extent of inequality aversion may depend on the domain in life considered (Tobin 1970): people might be averse to inequalities in income and health, but not with respect to education outcomes. One explanation for such differences might refer to the role of choice. Indeed, luck egalitarianism distinguishes the legitimacy of inequalities arising from unforeseen circumstances rather than choice (Barry 2006; Wikler 2002). Empirical evidence suggests that people tend to accept inequalities that are partly or fully a result of people’s choices (Schokkaert and Devooght 2003). So, people may differ in their sensitivity to inequality in different domains because of different perceptions in each domain and because unforeseen events may have different impacts in different domains. Furthermore, inequality issues may not be perceived in the same way in different societies (Gimpelson and Treisman 2018). Finally, another explanation for differences in inequality aversion is that different societies might be more or less averse to risk. If higher inequality entails a higher likelihood of ending up at the bottom of the distribution, then risk-averse people are likely to prefer allocations entailing lower inequality (Cowell and Schokkaert 2001; Amiel et al 2015).

The COVID-19 pandemic had far-reaching effects across Europe with the European Centre for Disease Prevention and Control (ECDC) reporting 4.2 million confirmed cases of the virus and over 196,000 deaths in the first year of the pandemic (ECDC 2020). It has given rise to declines in income and wealth (Hanspal et al. 2020),  strained government welfare programmes and increased economic anxiety (Bitler et al. 2020; Fetzer et al. 2021). The pandemic provides an opportunity to examine how inequality aversion changes in a time of crisis, paying attention to the role of risk aversion, and personal exposure to the health or economic consequences of the pandemic in shaping these behaviours. A recent review of the effect of viral pandemics suggests that it is not clear from the literature whether people become more tolerant and cooperative during a pandemic (Seitz et al. 2020). If following COVID-19 regulations steers some cooperation, both with other people and with the government for collective rather than purely individual benefit, it is reasonable to expect pro-sociality: people that care (empathise) about others, might become more sensitive to inequality. Consistently, evidence suggests that people who were more pro-social before the pandemic are more likely to engage in desirable health-related behaviours during the pandemic such as physical distancing, following hygiene recommendations, informing themselves about how they can help others; and donating financial resources towards efforts to fight COVID-19 (Campos-Mercade et al. 2020). Pro-social behaviours are therefore hypothesised to be an important determinant of inequality preferences.

In this paper we report the estimates from the elicitation of individual-level health- and income-inequality aversion and record health and employment shocks experienced during the first wave of the COVID-19 pandemic. Although this implies dropping some of the traditional assumptions of inequality-aversion experiments such as those made in veil-of-ignorance approaches (Costa-Font and Cowell 2019), earlier work suggests that the elicitation methods used in this study are consistent with the use of lottery-based methods that proxy veil of ignorance assumptions (Costa-Font and Cowell, 2022). We estimate how individual-level aversion to health and income inequality varies across Italy, Germany, and the United Kingdom, and whether inequality aversion is affected by health, income and employment shocks. This is important as people’s concern about inequality may depend on their position in terms of income, health status or employment with respect to others- in the group (Fehr and Schmidt 1999, 2003).^3^ Rather than relying on indirect elicitation techniques, that require people to make choices in hypothetical future scenarios (Costa-Font and Cowell 2019), we instead employ a direct trade-off elicitation technique, a widely used technique in identifying trade-offs in the health domain (Williams and Cookson 2006) in similar cardinal formats as those used here, as well as in choice and validation experiments (Arroyos-Calvera et al 2019; Dietz and Atkinson 2010).

The paper is organised as follows. Section 2 sets out the theoretical background and related literature. Section 3 describes the survey, elicitation strategy, and empirical specification. Section 4 contains the descriptive results for the three countries. Section 5 presents the results from regression analysis describing the impacts of health and employment shocks on health and inequality aversion conditional on controlling for key confounding variables such as income, education, demographics and especially risk aversion. Section 6 reports the results from a range of difference-in-differences strategies where we compare the impact of exposure to COVID-19 on inequality aversion in different risk groups in the United Kingdom; finally. Section 7 concludes.

## Background

### Theoretical background

The concept of inequality aversion (IA) can be applied to distributions in different *domains*, wealth, health and so on. To fix ideas, let us talk about “domain $d$” which could be any one of these; each person $i$ has an amount ${x}_{i}$ in this domain, the mean value of $x$ over the $n$ persons in society is $\mu$ and the inequality (according to some specified inequality measure) for the distribution in domain $d$ is $I$.

It is useful to contrast three different ways of thinking about IA. We could (1) think about IA in terms of the rate at which “society” is prepared to accept a reduction in $\mu$ in exchange for a reduction in $I$ (Atkinson 1970). We could instead (2) think about IA in terms of $i$’s *personal* evaluation; if $i$’s utility depends on ${x}_{i}$ and $I$ then IA can be seen as $i$’s evaluation of a personal trade-off between the two (Carlsson et al. 2005). Finally, we could (3) think about IA as $i$’s *social* evaluation; if $i$ has views on social aggregates then IA can be seen as $i$’s evaluation of a personal trade-off between $\mu$ and $I$.

Here we adopt approach (3): individual evaluation of social trade-offs between the mean of $x$ and the inequality of $x$. For present purposes we could use one of the simplest representations of these individual preferences about social choices:

$$\left[1-{\gamma }_{i}\right]\mathrm{log }\mu -{\gamma }_{i}\mathrm{log}I,$$

(or an increasing transformation of this) where ${\gamma }_{i}$ is a taste parameter that represents $i$’s implicit price of inequality reduction; if ${0<\gamma }_{i}<1$, then person $i$ is inequality averse. A one percent reduction in inequality is valued by $i$ as being worth a ${\gamma }_{i}/\left[1-{\gamma }_{i}\right]$ percent reduction in mean income: if ${\gamma }_{i}=0$ then $i$ gives priority to mean income and if ${\gamma }_{i}=1$ then $i$ gives priority to inequality.^4^

In this paper, we focus on the elicitation of the preference parameter ${\gamma }_{i}$ in the income domain and the health domain separately, the factors that appear to account for differences in ${\gamma }_{i}$ between subgroups of the population, and the role that exposure to COVID-19 may have had in shifting this parameter.

### Inequality aversion in income and health

Studies eliciting direct measures of income-inequality aversion differ in the methods used, and estimates can be heterogeneous when experimental methods are used. Leaky buckets experiments (examining the tolerance to transferring income from the rich to the poor) indicate values of inequality aversion close to zero (Amiel et al. 1999; Pirttila and Uusitalo 2010). However, methods based on eliciting direct preferences over alternative income distributions, typically in larger samples, using different elicitation techniques such as the imaginary grandchild, suggest estimates that are significntly larger (Johansson-Stenman et al. 2002; Carlsson et al. 2005).

To date, studies eliciting health-inequality aversion have not been conducted as extensively as they have in the income domain. In the context of attitudes towards the distribution of organ transplants, Ubel and Loewenstein (1996) showed evidence of a preference for an egalitarian equilibrium of giving everyone the chance of having a transplant rather than excluding those least likely to have a successful transplant. Leibler et al. (2009) found that support for a Pigou-Dalton transfer from the better off to the worse off was stronger in the income domain as opposed to the health domain. Consistent with these findings, Abásolo and Tsuchiya (2020) report survey evidence from Spain suggesting that income-inequality aversion is stronger than health-inequality aversion. Consistently, Hurley et al*.* (2020) estimate an income and health-inequality aversion measure for a sample of the general public in Ontario, Canada using a publicly representative online survey. They distinguish between bivariate inequality aversion and univariate inequality aversion that draws on comparable methods. They find evidence of strong income-inequality aversion and weaker aversion to health inequality and income-related health inequality. However, these studies provide data from only single countries, and do not attempt to examine changes over time, nor explore the impacts of shocks such as the employment and health shocks associated with COVID-19 on inequality aversion.

Despite substantial global policy concern about health inequality and universal health coverage (Marmot et al. 2012; Rodin and Ferranti 2012), individual health IA has been studied much less extensively than individual income IA and the two are rarely examined as distinct concepts (Abásolo and Tsuchiya 2013). Unlike income, health cannot be directly redistributed, it can be indirectly redistributed for example by influencing the priority or value (weights) assigned to recipients of health care programmes that in turn impacts the distribution of health outcomes. Hence, we can conceptualise health inequality in a similar way as income inequality, even though the way in which such inequality aversion would be factored in to reduce inequality might differ by domain.

## Data and methods

### Data

We draw on a set of comparable surveys to elicit IA estimates and to analyse the determinants of IA preferences. The surveys collect data that are representative of the populations in three countries: online interviews were carried out o 29th–31st January 2016 (UK only) and 15th–19th May 2020 amongst adults aged 16–75 in the UK and Germany, 16–70 in Italy.^5^ The English version of the 2020 questionnaire used is reproduced on the next page. This English questionnaire was translated into German and Italian for survey participants in the respective countries. Respondents of both surveys were part of a representative panel used regularly by market research companies to carry out survey research in every country. Questions in both years were piloted only after some preliminary analysis the rest of the sample was collected.

The 2020 survey was conducted during the initial stages of the COVID-19 pandemic in the UK, Germany, and Italy and adjusted the results for a range of interpersonal differences including: risk aversion (people who are risk loving tend to be less averse to inequality), income (people who are better off tend to be less averse to inequality), age (younger people are less averse to inequality), and education (better-educated people tend to be more averse to inequality).

The survey consisted of two groups of questions. The first group (Q1, Q2) concerns individual preferences for inequality in the health and income domains, and the second group (Q3–Q6) cover individual risk preferences and exposure to health, income, and employment shocks.^6^ In addition, information about gender, age and other personal characteristics was collected. The left-hand side of Table 1 gives the overall number of respondents and the breakdown by subgroups in the 2016 and 2020 subgroups. In each survey there were fewer female than male respondents, 70–75% were in the age range 25–64, and about half described themselves as being “medium” both in terms of educational attainment and in terms of income level. The following is the text of the questionnaire survey used in the United Kingdom survey where ‘don’t know’ and ‘preferred not to say’ where possible responses, and a similar text was used in the Germany and Italy surveys:

**Q1.** Would you say that reducing income inequality (income differences) in the United Kingdom is more or less important than improving its total national income?

Please read both statements and indicate your opinion on the following scale. The closer you place your answer to a statement the more it represents your opinion.

1 Reducing income inequality is more important than improving total national income.

…….

10 Improving total national income is more important than reducing income inequality.

**Q2.** Would you say that reducing the inequality (or individual differences) in life expectancy in the United Kingdom is more or less important than improving the average population life expectancy in the United Kingdom?

Please read both statements and indicate your opinion on the following scale. The closer you place your answer to a statement the more it represents your opinion.

1 Reducing inequality in life expectancy is more important than improving average population life expectancy.

…..

10 Improving average population life expectancy is more important than reducing inequality in life expectancy.

**Q3.** Are you generally a person who is willing to take risks or do you try to avoid taking risks?

Please answer on the following scale, where 1 is very unwilling to take risks and 10 is very willing to take risks.

1 – Very unwilling to take risks.

…

10 – Very willing to take risks.

**Table 1:** Descriptive statistics

			**2016**				
	**Country**			**Inequality, risk aversion (Q1, Q2, Q3)**			
UK	2008				${\gamma }^{y}$	${\gamma }^{h}$	$\rho$
				mean	0.528	0.450	0.545
				Low	5.8%	9.1%	3.4%
	**Age**			Medium	85.6%	85.4%	86.6%
< 25	185 (9%)			High	8.6%	5.4%	10.0%
25–64	1409 (70%)						
65+	414 (21%)			*N*	1943	1951	1979
	**Gender**						
Male	1120						
Female	888			**Shocks (Q4, Q5)**			
	**Education**	**Income**			*Health*	*Income*	
Low	60 (3%)	333 (18%)		No shock	1490	1653	
Medium	975 (49%)	981 (54%)		Minor	307	224	
High	939 (48%)	495 (27%)		Major	187	108	

			**2020**				
	**Country**			**Inequality, risk aversion (Q1, Q2, Q3)**			
UK	2295				${\gamma }^{y}$	${\gamma }^{h}$	$\rho$
Italy	2189			Mean	0.568	0.504	0.546
Germany	1202						
				Low	6.6%	8.4%	3.9%
	**Age**			Medium	78.1%	81.3%	86.5%
< 25	764 (13%)			High	15.3%	10.4%	9.6%
25–64	4232 (74%)						
65+	690 (12%)			*N*	6754	6549	7456
	**Gender**						
Male	2913 (51%)						
Female	2756 (49%)			**Shocks (Q4, Q5)**			
	**Education**	**Income**			*Health*	*Income*	*Employm't*
Low	629 (11%)	1598 (28%)		No shock	4627	2807	2538
Medium	3126 (55%)	2461 (43%)		Minor	502	1575	2665
High	1934 (34%)	927 (16%)		Major	414	1128	303

Note: ${\gamma }^{y}$, ${\gamma }^{h}$, $\rho$ mean, respectively, income-inequality aversion, health-inequality aversion, risk aversion

We would now like to ask you a couple of questions about you and your household’s health and financial situation since the start of the coronavirus pandemic.

These are not mandatory to answer and there is a ‘prefer not to say’ option available. If you do answer these questions, this information will be kept securely and will only be combined with other people’s answers when reporting the results so that you cannot be identified.

**Q4. **Have you or a member of your household suffered a medical emergency, in the last 3 months? Please select all that apply.

1. Yes, a minor medical emergency that did not require hospitalisation.
2. Yes, a major medical emergency that required hospitalisation
3. No medical emergency in the last 3 months
4. Prefer not to say.

**Q5. **Have you or a member of your household experienced any impact to finances in the last 3 months? Please select all that apply.

1. Yes, minor impact to finances.
2. Yes, major impact to finances.
3. No, there has been no change to finances.
4. Prefer not to say.

**Q6. **Still thinking about the last 3 months, which, if any, of the following describes your situation during this time? Please select all that apply.

1. I or a member of my household has had a temporary salary reduction, but still working.
2. I or a member of my household has been put on furlough.
3. I or a member of my household has been placed on temporary unpaid leave.
4. I or a member of my household has been made redundant.
5. I or a member of my household has temporarily closed my/their own business.
6. I or a member of my household has had to permanently close my/their own business.
7. My or a member of my household’s financial situation has changed for another reason.
8. None of these

### Methods

The main method here is an implementation of the approach described in questions reported in section (3.1) to elicit IA as outlined in Sect. 2.1. This is a contribution to the literature on the measurement of income inequality and to the broader literature in social science that has focused on health IA (Marmot et al. 2008; Lagomarsino et al. 2012). However, a version of this technique is widely used in the health economics literature to identify trade-offs between different dimensions of health and quality of life (Williams and Cookson 2006). Given that our purpose is to make sure our estimates are representative of the population, we have designed a direct elicitation trade off,^7^ we have simply extended the methods to reflect trade-offs between equity and efficiency in a way that is easily understood in a wide scale survey that is expected to be comprehensible by all population groups.

Although we do not elicit the value of ${\gamma }_{i}$ (introduced in Sect. 2.1) directly through experimental methods, questions 1 and 2 are used to elicit income and health IA by directly inviting responses about distributional judgments. The responses in Q1 and Q2 run from 1 (priority to inequality) to 10 (priority to the total income).^8^ So, to obtain the estimates of IA we transform the values using the formula ${\gamma }_{i}=\left[10-{q}_{i}\right]/9$ where ${q}_{i}$ is the chosen response to Q1 or Q2 respectively.

In a similar way the responses to Q3 can be transformed to provide a measure of risk aversion (RA): ${\rho }_{i}=\left[10-{q}_{i}\right]/9$ where ${q}_{i}$ is the chosen response to Q3.^9^

For both IA and RA special consideration is given to the extreme values where ${\gamma }_{i}$, ${\rho }_{i}$ take the values 0 or 1, as explained in Sect. 4 below. One potential concern is that a respondent may conceivably give a lower number (more inequality averse) for income than for health, not because they are more averse to income inequality but because they know that the income distribution is wider and more skewed. However, if the size of inequality in the country was driving the effect we should have higher income inequality in the UK. However, we find that Germany exhibits the highest inequality aversion. Additionally, whilst it might be the case that some respondents conflate inequality aversion and the perceived magnitude of inequality, the difference-in-difference estimates described in the following section should help to address these concerns. Furthermore, we have found that inequality estimates in 2016 correlate with other estimates that have been elicited from lottery-based measures (Costa-Font and Cowell 2022). That is, in an earlier study, Costa-Font and Cowell (2022) have elicited inequality aversion where individuals were requested to make choices between lotteries differing in inequality and average outcomes on behalf of an imaginary grandchild for both income and health. They found evidence of a high correlation between such measures and the inequality aversion measure used in this study, though the latter is less costly to retrieve in a representative survey.

Finally, we assume that other confounders are stable over time which is reasonable given the short period of time (shorter than five years) between the two data points of our study.

### Econometric model

Relatively little is known about factors that explain changes in inequality aversion attitudes, although it is plausible that changes in health or economic conditions affect the way that people view inequality and their preferences. Most evidence on the determinants of inequality aversion comes from small-scale experiments with limited external validity.^10^

The empirical approach involves specifying a model of ${\gamma }_{i}^{d}$, the inequality aversion of person $i$ in domain $d$, where $d$ is either $y$ (income) or $h$ (health). We have two main specifications, the results of which are presented in Sects. 5 and 6 respectively.

The basic specification is:

1 $${\gamma }_{i}^{d}={\alpha }_{0}+{\alpha }_{1}{s}_{i}+{\alpha }_{2}{C}_{k}+{\alpha }_{3}{X}_{i}+{\varepsilon }_{i},$$

where ${\gamma }_{i}^{d}$ is person $i$’s inequality aversion in dimension $d$, ${s}_{i}$ is a shock experienced by $i$, ${C}_{k}$ captures country-$k$ specific effects and ${X}_{i}$ captures the effects on $i$ of income, education, risk preferences, demographics and other variables that can affect inequality preferences.

Clearly, the time element is absent from Eq. (1), but the specification can be adapted to include the effects on IA resulting from vulnerability to COVID-19, and the effects of personal health and financial shocks observed in 2016 and in 2020. We can, of course, introduce ${\gamma }_{it}^{d}$ and ${X}_{it}$ as the time-varying counterparts of ${\gamma }_{i}^{d}$ and ${X}_{i}$; but we need to do more to construct a causal model.

This can be done by considering COVID-19 as a “treatment” that affects a subgroup of the target population.^11^ This subgroup may be defined in terms of experienced shocks or in terms of vulnerability (for example in terms of age). The general form of the required model is

2 $${\gamma }_{it}^{d}={\beta }_{0}+{{\beta }_{1}{P}_{t}+\beta }_{2}{T}_{it}+{\beta }_{3}{P}_{t}{T}_{it}+{\beta }_{4}{X}_{it}+{\varepsilon }_{it}$$

where ${P}_{t}$ is a *pandemic dummy* which takes the value 1 if the year is 2020 and 0 otherwise, and ${T}_{it}$ is a variable that specifies the treatment group, taking the value 1 if $i$ is in the group at time $t$ and 0 otherwise ($i$ is in the control group). In this case the treatment group consists of those who are “targeted” by Covid-19, so that ${T}_{it}$ can be expressed as

$${T}_{it}=\phi \left({{s}_{it},v}_{it}\right)$$

where ${s}_{it}$ is a *shock indicator* indicating whether person $i$ reported a specified shock at time $t$, and ${v}_{it}$ is a *vulnerability indicator* indicating whether $i$ belongs to a specified vulnerable group at time $t$.

To apply the difference-in-differences (DiD) method using Eq. (2) we need to make precise the specification of the indicators ${s}_{it}$, ${v}_{it}$ and the function $\phi$ used in determining the treatment group for the COVID-19 pandemic.^12^ For example, if membership of the COVID-19 treatment group requires *both* experiencing a shock *and* being in the vulnerable group, then we could just have $\phi \left({{s}_{it},v}_{it}\right)={{s}_{it}v}_{it}$. This issue is discussed further in Sect. 6.

## Results: descriptive evidence

### Overview

The right-hand side of Table 1 provides a first impression of the evidence on attitudes towards inequality and risk, elicited from Q1, Q2, Q3 and then captured by the individual values of the aversion parameters ${\gamma }_{i}^{y}$, ${\gamma }_{i}^{h}$, ${\rho }_{i}$.

It is clear from the rows labelled “mean” for 2016 and 2020 that, on average, IA is higher for income than for health, and that IA in each domain is higher in 2020 than in 2016. By contrast, RA in 2020 is approximately the same as in 2016. However, Table 1 reveals more about the patterns of IA and RA by summarising responses in three broad categories—Low, Medium and High. In each case the row labelled “High” refers to cases where the respondent chose ${q}_{i}=1$ from the 1–10 scale, which would then yield the extreme value ${\gamma }_{i}^{y}=1$ (in the case of Q1), or ${\gamma }_{i}^{h}=1$ (for Q2), or ${\rho }_{i}=1$ (for Q3).^13^ What is remarkable is that the proportion of respondents in the “High” IA category in 2020 is almost twice what it was in the 2016 sample. This applies to both dimensions, income, and health, but it does *not* apply to RA, where the proportion in the “High” category was slightly lower in 2020. Three questions arise, discussed in Sects. 4.2 to 4.4.

### Inequality aversion, risk aversion and risk

First, what could be behind this difference in IA? Although we do not measure the causes of inequality across domains in our elicitation method, at first glance it does not seem to be driven by a difference in risk *aversion*, so what about a perceived change in risk itself? A preliminary look at the information on shocks that were reported show that, in the 2016 sample, 15.5% of the sample reported a minor health shock and 9.4%; these proportions were lower in the 2020 sample (9% minor and 7.5% major). But the picture with income shocks is in sharp contrast: the proportion of the 2020 sample that experienced a shock is almost three times the proportion in the 2016 sample.

### Country breakdown in 2020

The second question is this: given that the 2020 sample covers three countries, what does the evidence on inequality-aversion and risk-aversion look like when we unpack the responses from the different national subsamples?^14^ The top half of Table 2 provides this information in the same format as the bottom half of Table 1.

**Table 2:** Inequality and risk aversion: country detail

	Inequality, risk aversion (by country, 2020)								
	UK			Italy			Germany		
	${\gamma }^{y}$	${\gamma }^{h}$	$\rho$	${\gamma }^{y}$	${\gamma }^{h}$	$\rho$	${\gamma }^{y}$	${\gamma }^{h}$	$\rho$
Mean	0.579	0.546	0.551	0.564	0.505	0.522	0.633	0.532	0.585
Low	6.7%	7.3%	3.8%	7.9%	9.3%	5.6%	5.3%	7.3%	1.8%
Medium	76.6%	79.1%	87.3%	74.5%	79.3%	84.3%	73.4%	80.8%	88.8%
High	16.7%	13.6%	8.9%	17.6%	11.5%	10.1%	21.3%	11.9%	9.5%

	Within-year correlations				
	UK 2016	UK 2020	It 2020	Ger 2020	All 2020
$\mathrm{corr}\left({\gamma }^{y},{\gamma }^{h}\right)$	0.563	0.571	0.588	0.422	0.558
$\mathrm{corr}\left({\gamma }^{y},\rho \right)$	0.169	0.193	0.227	0.092	0.185
$\mathrm{corr}\left({\gamma }^{h},\rho \right)$	0.113	0.134	0.189	0.087	0.137

Note: ${\gamma }^{y}$, ${\gamma }^{h}$, $\rho$ mean, respectively, income-inequality aversion, health-inequality aversion, risk aversion

Begin with inequality aversion. In 2020, for each country ${\overline{\gamma }}^{y}$, the average value of income-inequality aversion, is higher than ${\overline{\gamma }}^{h}$, the average value of health-inequality aversion); remarkably the ${\overline{\gamma }}^{y}- {\overline{\gamma }}^{h}$ gap for Germany is twice as large as that in Italy.^15^ The Germans emerge as the most income-inequality averse of the three countries in terms of the mean value ${\overline{\gamma }}^{y}$ and also in terms of the proportion of respondents in the “High” category (where ${\gamma }^{y}=1$). Table 2 also shows that the UK is the most health-inequality averse of the three countries examined, again in terms of the mean value ${\overline{\gamma }}^{h}$ and in terms of the proportion of respondents in the “High” category (where ${\gamma }^{h}=1$).

We can also use Table 2 to compare the UK with itself: Comparing the UK situation in 2016 (top right of Table 1) and the situation in 2020 (top left of Table 2). We find that the difference in ${\overline{\gamma }}^{h}$ is about twice the difference in ${\overline{\gamma }}^{y}$; the proportion of those reporting “High” inequality aversion (where ${\gamma }^{y}$ or ${\gamma }^{h}=1$) increased much more in the case of health than for income. This suggests that circumstances of 2020 may have had a stronger effect on health-inequality aversion than on income-inequality aversion in the UK.

Now, we  look at the estimates of risk aversion, $\rho$. Table 2 shows that, as with income-inequality aversion, Germany is the most risk averse, followed by the UK; this is also borne out by the proportion of respondents in the “Low” category (where $\rho =0$) in each country: Italy has the lowest proportion of low-RA respondents, followed by the UK. Again, comparing the situation of UK 2016 with that of UK 2020 we find that: the mean value of $\rho$ increased slightly, but the proportion of respondents in the “High” category (where $\rho =1$) is *lower* in 2020. This is in sharp contrast to what appears to have happened to inequality aversion, in either domain.

**Fig. 1:**
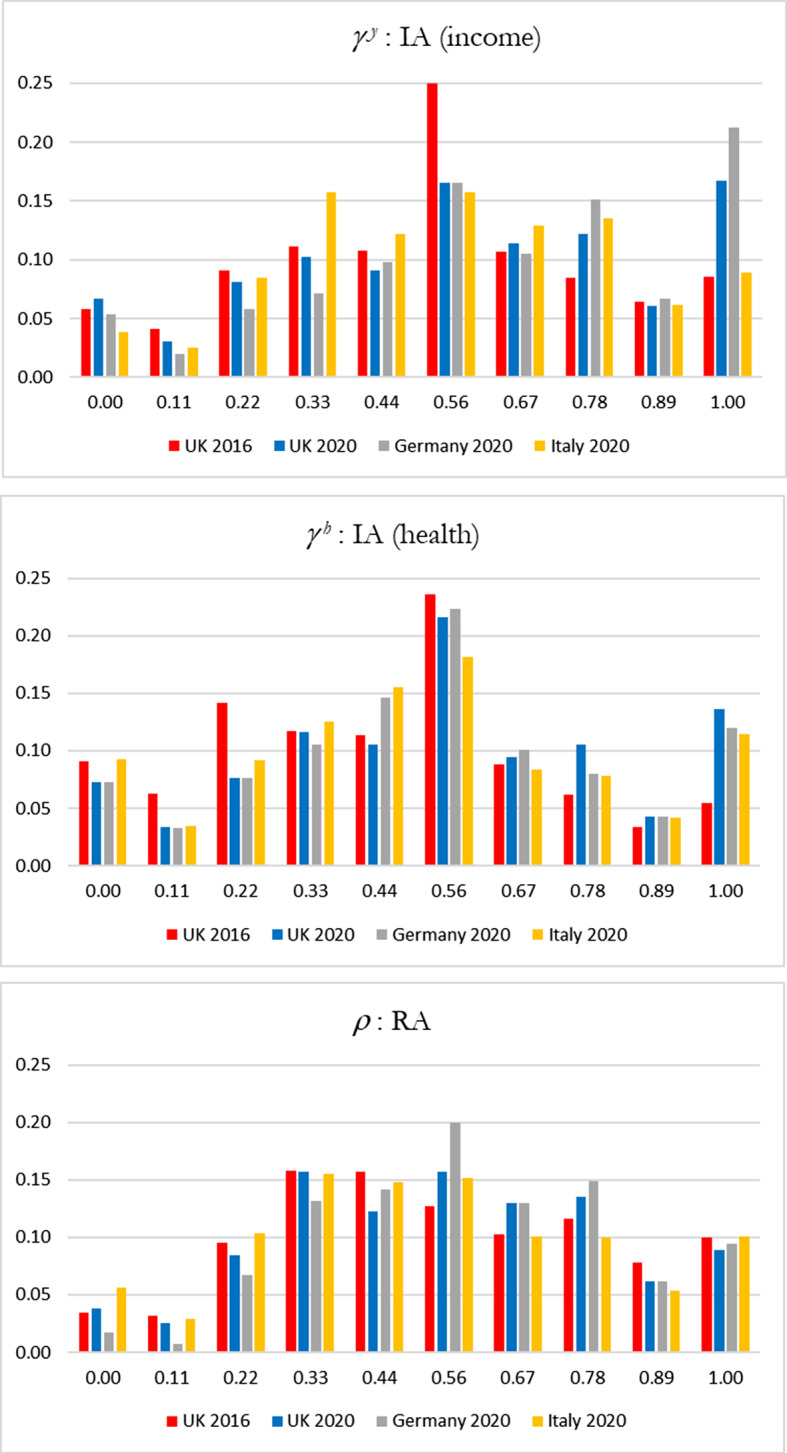
Inequality aversion and risk aversion (share of respondents in a 0–1 scale). Vertical axis refers to the share if responses in a 0 to 1 scale, and horizontal axis refers to the values

Figure 1 shows the detail of the distributions of ${\gamma }^{y}$, ${\gamma }^{h}$, $\rho$ derived from the responses from questions 1 to 3 in the survey. In the first two panels of Fig. 1 we compare the red bars (UK 2016) with the blue (UK 2020): the shift of observations from the mid-range of $\gamma$ in 2016 to $\gamma =1$ in 2020 is dramatic. Also clear is the contrast in Germany 2020 between the distribution of ${\gamma }^{y}$ (first panel) and the distribution of ${\gamma }^{h}$ (second panel). A further point is evident when we compare these two panels with the third panel showing the distribution of $\rho$: one is struck by the similarity of the height of the red and blue bars at each of the ten values of $\rho$ indicating that the distribution of estimated risk aversion in 2020 is much the same as in 2016. The contrast in the pictures for  $\gamma$ and the picture for $\rho$ is striking and reinforces the view that the difference in inequality aversion between 2016 and 2020 is not principally attributable to a change in risk aversion. One issue discussed in the literature is whether the distribution of inequality preferences is multimodal. However, Fig. 1 does not reveal a multimodal distribution, instead evidence suggests a single mode corresponding to focal point in responses at the value “5” and, in some cases, a secondary concentration at the upper end of the response distribution.

### Types of inequality aversion

The third question that arises is this: is there strong correlation between types of aversion? Are the high IA people the same ones in both income and health domains? Are high risk-aversion people also high inequality-aversion people? The lower part of Table 2 addresses this. For the UK (both 2016 and 2020) and Italy the correlation between ${\gamma }^{y}$ and ${\gamma }^{h}$ (individual inequality aversion in the two domains) lies between 0.56 and 0.59; in Germany it is somewhat lower. But the correlation between individual inequality aversion (in either domain) or risk aversion is much lower, for all countries, further estimates are provided in the online appendix as follows Appendix A refers to further descriptive statistics, Appendix B refers to the further regression estimates, and Appendix C reports the estimates of a fully specified difference-in-differences model.

## Regression evidence of inequality aversion

Next, we use a cross-section regression analysis based on Eq. (1) to examine further the descriptive results reported in Sect. 4. For the cross-sectional analysis Eq. (1) is estimated separately for 2016 and 2020. The summary of the coefficient estimates is reported in Table 3. Fully specified regressions are found in the Appendix in Tables is B1 to B4 for cross-section regressions, and Tables C1 to C8 for difference-in-differences regressions.

**Table 3:** Determinants of health- and income-inequality aversion

	Health-inequality aversion 2020	Income-inequality aversion 2020	Health-inequality aversion 2016	Income-inequality aversion 2016
**Age group (reference: 25–64)**				
< 25	− 0.0283** (0.0141)	− 0.0237* (0.0143)	− 0.0217 (0.0238)	0.00330 (0.0239)
65+	0.00694 (0.0142)	0.0154 (0.0142)	− 0.0205 (0.0167)	− 0.0276* (0.0167)
**Gender (reference: female)**				
Male	− 0.00671 (0.00917)	0.00270 (0.00921)	0.0372*** (0.0132)	0.0235* (0.0131)
**Education level (reference: medium)**				
Low education	− 0.0171 (0.0164)	− 0.0235 (0.0165)	0.107** (0.0418)	0.0944** (0.0413)
High education	0.0416*** (0.00995)	0.0327*** (0.0100)	0.0465*** (0.0135)	0.0453*** (0.0135)
**Income level (reference: medium)**				
Low income	0.0215** (0.0106)	0.0196* (0.0107)	− 0.0156 (0.0176)	0.0166 (0.0176)
High income	− 0.0296** (0.0122)	− 0.0347*** (0.0123)	− 0.0444*** (0.0155)	− 0.0566*** (0.0154)
**Risk aversion (0–1 scale)**				
	0.156*** (0.0181)	0.210*** (0.0181)	0.127*** (0.0248)	0.185*** (0.0248)
**Country (reference: UK)**				
Italy	− 0.0317*** (0.0103)	0.00467 (0.0104)		
Germany	− 0.0144 (0.0127)	0.0486*** (0.0127)		
**Health shock (reference: none)**				
Minor health shock	− 0.0337** (0.0155)	− 0.0237 (0.0157)	− 0.0146 (0.0182)	− 0.0309* (0.0182)
Major health shock	− 0.0327* (0.0173)	− 0.0437** (0.0178)	− 0.0167 (0.0230)	− 0.0419* (0.0229)
**Income shock (reference: none)**				
Minor income shock	0.0238* (0.0136)	0.0217 (0.0136)	− 0.00233 (0.0210)	− 0.00259 (0.0209)
Major income shock	0.0225 (0.0154)	0.0218 (0.0155)	0.0199 (0.0284)	0.0207 (0.0283)
**Employment shock (reference: none)**				
Temporary employment shock	− 0.0184 (0.0132)	− 0.0157 (0.0132)		
Permanent employment shock	− 0.00645 (0.0227)	− 0.0417* (0.0229)		
Constant	0.454*** (0.0169)	0.457*** (0.0170)	0.361*** (0.0206)	0.418*** (0.0206)
Observations	3982	4163	1734	1730
R-squared	0.039	0.055	0.029	0.050

Note: Standard errors in parentheses and robust standard errors. ***p < 0.01, **p < 0.05, *p < 0.1

### Baseline

We take the “baseline case” to be a middle-aged, middle-income, medium-educated, risk-neutral, female respondent in the UK with no reported shocks. To interpret the coefficient estimates of the regression reported in Table 3, recall the definition of inequality aversion in Sect. 2 and the description in Sect. 4 of the method of using the survey responses to compute ${\gamma }^{h}$ (IA, health domain) and ${\gamma }^{y}$ (IA, income domain). For each domain, the notional scale of ${\gamma }^{h}$ and ${\gamma }^{y}$ runs from 0 (indifference to inequality) to 1 (total priority to inequality)—as does the scale of risk-aversion $\rho$.

In 2020, for either domain, a person fitting the profile of the baseline case would have displayed inequality aversion approximately in the middle of this range with ${\gamma }^{h}=0.454$ and ${\gamma }^{y}=0.457$ (see the constant term in each of the two left-hand columns). However, these figures represent a considerable increase on the IA-values that the baseline person would have displayed in 2016: ${\gamma }^{h}=0.360$ and ${\gamma }^{y}=0.418$ (see the two right-hand columns).

We proceed from the baseline case by examining the apparent impact on estimates of ${\gamma }^{h}$ and ${\gamma }^{y}$ arising from (1) risk-aversion and shocks, (2) personal characteristics, (3) country characteristics.

### Risk aversion and shocks

It is clear from rows 6 and 7 of Table 2 that, as risk aversion increases, so does inequality aversion: this result is to be expected (Amiel et al. 2001; Cowell and Schokkaert 2001); it applies to both health and income domains, and it applies in both years.

The effect of shocks is more nuanced (see the lower part of Table 3). Wherever the coefficient is significantly different from zero a health shock is associated with a lower estimated IA, in both income and health domains. However, only rarely is there a significant effect on IA from income shocks (positive, in the health domain 2020) or from employment shocks (negative, in the income domain 2020).

### Personal characteristics

We identify a clear age effect in the 2020 sample. Notice that the coefficient on under 25’s (or “< 25”) is significant in both domains. One might be tempted to draw the conclusion “the older you are more the more inequality-averse you are.” But this only applies to 2020 and only to a comparison between youth and the reference group—there is no effect from being elderly. However, in 2016 there is, in the income domain, an effect in the opposite direction—suggesting that elderly respondents are less inequality averse than the reference group.

Although there is no significant difference between males and females in the 2020 sample, in the 2016 sample, males are more inequality-averse in terms of both health and income.

Having education higher than the reference level makes a person more inequality-averse in both domains and in both years of observation. Having education lower than the reference level has no significant effect on measured IA in 2020 but, again, has a positive effect on IA in 2016. On the other hand, the story with respect to income is simpler: having high income makes a person less inequality averse and (for 2020 only) having low-income results in higher inequality aversion. Roughly speaking, the more income you have the less you are concerned about inequality.

### Country subsamples

The contrast in IA between countries has already been glimpsed in Table 2. The rows labelled “Country” reveal the following for the 2020 sample.

In terms of health-inequality aversion there is no significant difference between the reference case in the UK and someone in Germany but switching from the reference case to Italy would lower the estimate of ${\gamma }^{h}$ by 0.031.

Finally, in terms of income-inequality aversion there is no significant difference between the reference case in the UK and someone in Italy but switching from the reference case to Germany would raise the estimate of ${\gamma }^{y}$ by 0.048.^16^

## A difference-in-differences approach

Although Sect. 5 showed that individuals experiencing health or employment shocks in the household during the COVID-19 pandemic tended to be significantly less inequality averse, this result might not be specific to a COVID-19 shock. So, to compare the effect of exposure to COVID-19 to similar health shocks pre-COVID, in this section we examine inequality preferences over time in the UK using a difference-in-differences specification.

### Specification of the treatment group

The implementation of Eq. (2) in modelling the COVID “treatment” requires further consideration of three things:

- *Shocks* ${s}_{it}$. COVID related shocks potentially include both reported health shocks and reported income shocks. Shocks could be minor, major or both.
- *Individual vulnerability* ${v}_{it}$. This is based on the risk that a person might contract COVID-19, considering his/her personal characteristics. The characteristics that are appropriate to our problem are, in the first place, being in the older age groups and secondly, living in a high-risk region. In what follows we distinguish between single-source and multi-source vulnerability.
- *The function* $\phi$. This can be chosen to be responsive either to ${s}_{it}$ or $\mathrm{to }{v}_{it}$ or to both, giving us alternative variants of the treatment group.

### The standard model

The principal focus will be on a *standard treatment group* characterised by the experience of health shocks and single-source vulnerability; the vulnerable are taken to be those aged over 65. The function $\phi$ is a simple multiplicative form so that:

$${T}_{it}={{s}_{it}v}_{it}$$

Within this framework we can consider further sub-variants of the model by allowing for flexibility concerning the conditioning covariates ${X}_{it}$. We have the option of including or omitting other personal characteristics and risk attitudes in the DID Eq. (2). In effect we may choose to constrain the parameter ${\beta }_{4}$ to be zero.

The model is estimated for the two UK samples (2016 and 2020) and the principal results for the standard treatment group^17^ are presented in Table 4 (which includes a fully specified model with and without controls).^18^

**Table 4:** DiD regressions for standard treatment group (UK 2016 and 2020)

	(1)	(2)	(3)	(4)
	${\gamma }^{h}$	${\gamma }^{h}$	${\gamma }^{y}$	${\gamma }^{y}$
${P}_{t}$	0.0936*** (0.009)	0.0981*** (0.0095)	0.0484*** (0.0091)	0.0472*** (0.0095)
${{s}_{it}v}_{it}$	− 0.0474 (0.0472)	− 0.0234 (0.0523)	− 0.0765* (0.0464)	− 0.0693 (0.0514)
${P}_{t}{{s}_{it}v}_{it}$	0.239*** (0.0840)	0.206** (0.0876)	0.200** (0.0848)	0.198** (0.0860)
**Conditioning covariates**				
Age < 25		− 0.0392*** (0.0144)		− 0.0286* (0.0146)
Age 65 +		− 0.0202 (0.0132)		− 0.0217* (0.0132)
Male		0.000183 (0.00949)		− 0.00834 (0.00952)
Low education		− 0.00855 (0.0240)		− 0.000319 (0.0242)
High education		0.0591*** (0.00978)		0.0552*** (0.00980)
Low income		− 0.00363 (0.0119)		0.0105 (0.0120)
High income		− 0.0397*** (0.0113)		− 0.0447*** (0.0114)
$\rho$		0.123*** (0.0182)		0.194*** (0.0183)
**Constant**	0.451*** (0.00632)	0.371*** (0.0151)	0.530*** (0.00648)	0.417*** (0.0152)
Observations	3846	3494	3916	3569
R-squared	0.031	0.060	0.009	0.056

Note: Standard errors in parentheses and robust standard errors. ***p < 0.01, **p < 0.05, *p < 0.1

### Health-inequality aversion

Columns 1 and 2 of Table 4 summarise the key results for ${\gamma }^{h}$. Recall that ${\gamma }^{h}$ has a minimum of 0 and a maximum of 1. We see from column 1 that, if conditioning covariates are excluded from the estimation, there is an increase of 0.24 in health-inequality aversion for people that experienced a health shock in 2020 and were part of a high COVID-19 risk age group compared to people in a high-risk age group that exhibited a health shock in 2016. The effect is slightly less (0.21) if conditioning covariates are included in the estimation.

### Income-inequality aversion

The results for ${\gamma }^{y}$ (also measured on a [0,1] scale) reveal a similar picture to the results for ${\gamma }^{h}$—see columns 3 and 4 of Table 4. Comparing people who experienced a health shock in 2020 and were part of a high-risk age group with people in similar circumstances in 2016, column 3 shows an increase of about 2.0 in income-IA. This effect is only very slightly reduced if conditioning covariates are included in the estimation (column 4).

## Conclusion

Individual inequality aversion (IA) is important for understanding both how people perceive inequality, and their public priorities concerning distribution of relevant outcomes, especially in the income and health domain. Understanding IA in a time of crisis is crucial for public policy making.

Accordingly, we have focused on individual IA in terms of income and in terms of health in Germany, Italy and the UK during the first year of the COVID-19 pandemic. For the UK similar estimates were also produced for 2016 and are used to identify the impact of the pandemic on inequality aversion.

Cross-sectional analysis shows the following. First, in each subsample people are more inequality averse with respect to income than health. Second, people in the UK were more inequality averse in 2020 than in 2016, with the difference in IA for health twice that for income. Third, in all three countries, being risk-loving and having a higher income are associated with significantly lower levels of IA; but being older and having more education is associated with higher levels of IA. Fourth, people experiencing health or employment shocks during the COVID-19 pandemic were less averse to health and income inequality; but experiencing a similar shock in 2016 did not significantly modify health-IA estimates. This is consistent with evidence suggesting a reduction of individuals empathy during a catastrophe such as a pandemic (Seitz et al 2020).

Using a difference-in-differences model for the UK, we find that people who were in high COVID-19 risk groups (age 65 + and in a high-risk region) and who at the time experienced a health shock during the pandemic displayed significantly higher level of both health IA and income IA than similar people in 2016. These effects are not driven by a change in innate risk aversion^19^ but may have been attributable to the changed circumstances specific to the pandemic.

## Supplementary Information

Below is the link to the electronic supplementary material.

Supplementary file1 (DOCX 96 kb)

## Data Availability

The data used in this paper can be made available upon the author’s request.

## Footnotes

1 See Amiel et al. (1999), Pirttila and Uusitalo (2010), Johansson-Stenman et al. (2002), Carlsson et al. (2005).

2 In arguing the basis for inequality-averse social preferences Tricomi et al. (2010) find neural evidence suggesting that highly paid people exhibit higher gains from paying others as compared to paying themselves.

3 Fehr and Schmidt (1999) experiment examine the distributional effect resulting from unearned income ignoring no pre-redistribution earnings.

4 One could use any cardinalisation involving a monotonic transformation of this log-linear formula. We have used this cardinalisation to make the connection to the empirical application in Sect. 3 clear. In Sect. 3 we report the elicitation of respondents’ views on a scale from 1 to 10, so that the aversion parameter is simply rescaled. Notice that this log-linear formula is easily reinterpreted for the other approaches to IA. For approach (1) ${\gamma }_{i}$ is restricted to being the same value for all $i$. For version (2) we replace the argument $\mu$ by ${x}_{i}$.

5 The surveys were carried out by ICM Unlimited (2016) and IPSOS-MORI (2020).

6 The 2016 survey included the same questions as the one for 2020 but did not ask about employment shocks.

7 Previous research (Costa-Font and Cowell 2022) has shown that this direct elicitation technique elicits preferences that are consistent with implicit trade-offs.

8 The scale treated the scale as continuous for simplicity.

9 The measure of risk aversion employs a different framing than the inequality aversion questions, the reason being that the exact measure of risk aversion has been validated against lottery based methods of risk aversion elicitation (Dohmen et al 2017).

10 Examples of previous research using small-scale experiments include Amiel and Cowell (1999), Bolton and Ockenfels (2000), Bosmans and Schokkaert (2004), Carlsson et al. (2005), Cowell and Schokkaert (2001), Fehr and Schmidt (1999).

11 Several papers have used this type of approach to model the effect of some type of natural disaster. See, for example, Behrman and Weitzman (2016), Rodríguez-Oreggia et al. (2008)

12 The importance of using a difference-in-differences specification lies in that it allows computing the difference between treatment and control before and after the pandemic, netting out such country or group specific individual differences.

13 Likewise “Low” refers to cases where the respondent chose ${q}_{i}=10$, which would then yield the extreme value ${\gamma }_{i}^{y}=0$ (in the case of Q1), or ${\gamma }_{i}^{h}=0$ (for Q2), or ${\rho }_{i}=0$ (for Q3); “Medium” refers to cases where the respondent chose ${q}_{i}=\mathrm{2,3},\dots ,9$ which yields an aversion parameter lying strictly between 0 and 1.

14 A more detailed breakdown is given in Appendix A.

15 For pre-COVID19 evidence see Hurley et al. (2020).

16 Appendix B provides detailed examination of alternative specifications of the regression Eq. (1)

17 For an exhaustive treatment of alternate specifications see Appendix C. The four columns of Table 4 are based on Tables C2, C4, C6 and C8 in Appendix C.

18 Other specifications considering alternative treatments are reported in the appendix, and generally refer to other potential treatment effects which did not reveal different treatment effects.

19 As we saw in Tables 1 and 2, the mean value of $\rho$ in the UK sample was 0.545 in 2016 and 0.551 in 2020. For the treatment group t the mean value of $\rho$ was 0.614 in 2016 and 0.628 in 2020: so although risk aversion in the treatment group was significantly higher than in the UK as a whole the difference between the 2016 and the 2020 values of $\rho$ was still modest.
