# Supplementary material for: How does exposure to COVID-19 influence health and income inequality aversion?
Source: Soc Choice Welfare. 2023 May 19:1–23. Online ahead of print. doi: 10.1007/s00355-023-01460-8 (PMC10197041; doi:10.1007/s00355-023-01460-8)
Supplement: Supplementary file 1 — Supplementary file1 (DOCX 96 kb) [file 355_2023_1460_MOESM1_ESM.docx]

# Online Appendix

# Appendix A: Descriptive Statistics -- detail

A comparison of estimates across age groups (Tables A1 and A2), shows that the differences described in the text are primarily driven by older and middle-aged people. Income-inequality aversion is larger amongst older and middle-aged people in Germany than the corresponding groups in Italy and the UK; there are no significant cross-county differences in health-inequality aversion. There is little evidence to suggest any gender differences in inequality aversion in either domain (Tables A3, A4 and A5).

Figures A1 – A6 provide graphical evidence of differences in health- and income-inequality aversion in the UK by age, income group and gender in 2016 and 2020. They suggest lower levels of inequality aversion among younger people and higher income respondents**.**

**Table A1: Within-country age differences in inequality aversion**

|  | Obs (A) | Obs (B) | t value |
| --- | --- | --- | --- |
| **Income IA – Germany** |  |  |  |
| A=Middle – B=Older Age | 464 | 159 | -2.9** |
| A=Young – B=Older Age | 350 | 159 | -4.85*** |
| A= Young– B=Middle Age | 350 | 464 | -3.05*** |
| **Health IA- Germany** |  |  |  |
| Middle – Older Age | 428 | 147 | -.8 |
| Young – Older Age | 329 | 147 | -1.25 |
| Young – Middle Age | 329 | 428 | -.7 |
| **Income IA – Italy** |  |  |  |
| Middle – Older Age | 1029 | 115 | -.8 |
| Young – Older Age | 721 | 115 | -2.2** |
| Young – Middle Age | 721 | 1029 | -2.95*** |
| **Health IA – Italy** |  |  |  |
| Middle – Older Age | 993 | 112 | -2** |
| Young – Older Age | 694 | 112 | -2.75*** |
| Young – Middle Age | 694 | 993 | -1.7 |
| **Income IA – UK** |  |  |  |
| Middle – Older Age | 892 | 259 | 1.35 |
| Young – Older Age | 822 | 259 | -1.35 |
| Young – Middle Age | 350 | 464 | -3.05 |
| **Health IA – UK** |  |  |  |
| Middle – Older Age | 842 | 241 | 1.95 |
| Young – Older Age | 812 | 241 | -1.05 |
| Young – Middle Age | 812 | 842 | -4.5** |

Note: Standard errors in parentheses *** p<0.01, ** p<0.05, * p<0.1

**Table A2: Between-country age differences in inequality aversion**

|  | Obs 1 | Obs 2 | t value |
| --- | --- | --- | --- |
| **Older** |  |  |  |
| *Income IA Italy – Germany* | 115 | 159 | -3.4 |
| *Income IA UK – Germany* | 259 | 159 | -4.95 |
| *Income IA UK – Italy* | 259 | 115 | -.75 |
| *Health IA Italy – Germany* | 112 | 147 | .4 |
| *Health IA UK – Germany* | 241 | 147 | -.55 |
| *Health IA UK – Italy* | 241 | 112 | -.9 |
| **Middle** |  |  |  |
| *Income IA Italy – Germany* | 1029 | 464 | -3.9 |
| *Income IA UK – Germany* | 892 | 464 | -2.3 |
| *Income IA UK – Italy* | 892 | 1029 | 1.95** |
| *Health IA Italy – Germany* | 993 | 428 | -1.4 |
| *Health IA UK – Germany* | 842 | 428 | 2.7 |
| *Health IA UK – Italy* | 842 | 993 | 5.05 |
| **Young** |  |  |  |
| *Income IA Italy – Germany* | 721 | 350 | -2. |
| *Income IA UK – Germany* | 822 | 350 | -1.65 |
| *Income IA UK – Italy* | 822 | 721 | .9 |
| *Health IA Italy – Germany* | 694 | 329 | -1.7 |
| *Health IA UK – Germany* | 812 | 329 | -.2 |
| *Health IA UK – Italy* | 812 | 694 | 1.9 |

Standard errors in parentheses *** p<0.01, ** p<0.05, * p<0.1

**Table A3. Within-country gender differences in IA**

|  | Obs 1 | Obs 2 | t value |
| --- | --- | --- | --- |
| **Germany Income IA Female-Male** | 462 | 511 | -0.55 |
| **Germany Health IA Female-Male** | 419 | 485 | 0.7 |
| **Italy Income IA** Female-Male | 903 | 962 | -1.45 |
| **Italy Health IA** Female-Male | **868** | **931** | **-0.5** |

Standard errors in parentheses *** p<0.01, ** p<0.05, * p<0.1

**Table A4: Between-country Gender differences in IA**

| **Female** | **obs1** | **obs2** |  | **t- value** |  |
| --- | --- | --- | --- | --- | --- |
| **Income IA** Italy-Germany | 903 | 462 |  | -4.35 |  |
| **Health IA** Italy-Germany | 868 | 419 |  | -2.25 |  |
|  |  |  |  |  |  |
| **Male** | **obs1** | **obs2** |  | **t- value** |  |
| **Income IA** Italy-Germany | 962 | 511 |  | -3.95 |  |
| **Health IA** Italy-Germany | 931 | 485 |  | -1.1 |  |

Note: Standard errors in parentheses *** p<0.01, ** p<0.05, * p<0.1

**Table A5: Inequality Aversion by Gender**

|  | **Male** | |  | | **Female** | |  | |  |
| --- | --- | --- | --- | --- | --- | --- | --- | --- | --- |
| 2016 | | Mean | | St.Dev | | Mean | | St.Dev | |
| Aversion to Income | | 0.526 | | 0.261 | | 0.530 | | 0.275 | |
| Aversion to Health | | 0.437 | | 0.267 | | 0.465 | | 0.264 | |
| Income Shock (percentage) | | .153 | | .361 | | .178 | | .383 | |
| Health Shock (percentage) | | .236 | | .425 | | .259 | | .439 | |
| 2020 | | Mean | | St.Dev | | Mean | | St.Dev | |
| Aversion to Income | | 0.58 | | 0.295 | | 0.578 | | 0.302 | |
| Aversion to Health | | 0.536 | | 0.281 | | 0.516 | | 0.291 | |
| Income Shock (percentage) | | .509 | | .5 | | .521 | | .5 | |
| Health Shock (percentage) | | .17 | | .375 | | .165 | | .372 | |

**Figure A1: Income and income-inequality aversion in 2016**

Note: mean estimates of income and health-IA in the UK 2016

**Figure A2: Income and income-inequality aversion in 2020**

Note: mean estimates of income and health-IA in the UK 2020

**Figure A3: Income and health-inequality aversion by income group 2016**

**Figure A4: Income and health-inequality aversion by income group 2020**

**Figure A5: Income and health-inequality aversion by gender 2016-2020**

# Appendix B: Cross-sectional regressions -- detail

This appendix reports results from alternative specifications for the econometric model discussed in section 5 of the main text.

Table B1 reports results from alternative specifications for health-inequality aversion in 2020. Again, risk-loving people exhibit significantly lower inequality aversion, for all the different specifications. Specifications 4 to 9 include education and income. High-level education always increases inequality aversion higher income always reduces IA: this applies across all specifications. Health shocks reduce average health-inequality aversion. Similar results for health-inequality aversion in the UK in 2016 can be found in Table B2.

Results from alternative specifications for income-inequality aversion in the 2020 sample and the 2016 (UK) sample can be found in tables B3 and B4 respectively. Compared to the UK, income-inequality aversion is significantly higher in Germany, and the estimates are robust across different specifications. As with health-inequality aversion, income-inequality aversion is consistently lower among people under 25, and higher among people over 65. As with health-inequality aversion, we find no evidence of gender effects. Income and education effects are consistent with previous estimates for health-inequality aversion. Lower-income people are *more* inequality averse. In contrast, higher income people are less inequality averse. Major health shocks reduce income-inequality aversion. However, whilst income shocks do not modify inequality aversion estimates, permanent employment shocks reduce income-inequality aversion.

**Table B1: Health IA – 2020**

|  | (1) | (2) | (3) | (4) | (5) | (6) | (7) | (8) | (9) |
| --- | --- | --- | --- | --- | --- | --- | --- | --- | --- |
| **Age (reference: 25-64)** |  |  |  |  |  |  |  |  |  |
| <25 |  | -0.0480*** | -0.0355*** | -0.0362*** | -0.0294** | -0.0335** | -0.0322** | -0.0297** | -0.0283** |
|  |  | (0.0124) | (0.0124) | (0.0134) | (0.0138) | (0.0137) | (0.0137) | (0.0140) | (0.0141) |
| 65+ |  | 0.0219* | 0.00841 | 0.00804 | 0.00724 | 0.0105 | 0.00661 | 0.00654 | 0.00694 |
|  |  | (0.0130) | (0.0130) | (0.0138) | (0.0138) | (0.0139) | (0.0141) | (0.0142) | (0.0142) |
| **Gender (ref: female)** |  |  |  |  |  |  |  |  |  |
| Male |  | -0.0204** | -0.00838 | -0.00742 | -0.00559 | -0.00590 | -0.00868 | -0.00714 | -0.00671 |
|  |  | (0.00845) | (0.00847) | (0.00896) | (0.00905) | (0.00904) | (0.00906) | (0.00913) | (0.00917) |
| **Education (ref: medium)** |  |  |  |  |  |  |  |  |  |
| low education |  |  |  | -0.0152 | -0.0189 | -0.0157 | -0.0131 | -0.0184 | -0.0171 |
|  |  |  |  | (0.0160) | (0.0162) | (0.0162) | (0.0162) | (0.0164) | (0.0164) |
| high education |  |  |  | 0.0419*** | 0.0429*** | 0.0429*** | 0.0415*** | 0.0418*** | 0.0416*** |
|  |  |  |  | (0.00977) | (0.00985) | (0.00985) | (0.00986) | (0.00992) | (0.00995) |
| **Income (ref: medium)** |  |  |  |  |  |  |  |  |  |
| low income |  |  |  | 0.0217** | 0.0218** | 0.0211** | 0.0217** | 0.0223** | 0.0215** |
|  |  |  |  | (0.0103) | (0.0105) | (0.0105) | (0.0105) | (0.0106) | (0.0106) |
| high income |  |  |  | -0.0274** | -0.0303** | -0.0285** | -0.0292** | -0.0307** | -0.0296** |
|  |  |  |  | (0.0120) | (0.0121) | (0.0121) | (0.0121) | (0.0122) | (0.0122) |
| **Risk aversion** |  |  |  |  |  |  |  |  |  |
|  |  |  | 0.155*** | 0.158*** | 0.154*** | 0.159*** | 0.156*** | 0.154*** | 0.156*** |
|  |  |  | (0.0166) | (0.0176) | (0.0178) | (0.0178) | (0.0178) | (0.0180) | (0.0181) |
| **Country (ref: UK)** |  |  |  |  |  |  |  |  |  |
| Italy | -0.0412*** | -0.0405*** | -0.0367*** | -0.0309*** | -0.0303*** | -0.0309*** | -0.0300*** | -0.0308*** | -0.0317*** |
|  | (0.00942) | (0.00949) | (0.00943) | (0.0101) | (0.0102) | (0.0102) | (0.0102) | (0.0103) | (0.0103) |
| Germany | -0.0142 | -0.0154 | -0.0212* | -0.0168 | -0.0165 | -0.0146 | -0.0154 | -0.0152 | -0.0144 |
|  | (0.0116) | (0.0116) | (0.0115) | (0.0125) | (0.0126) | (0.0126) | (0.0126) | (0.0127) | (0.0127) |
| **Hlth. shock (ref:none)** |  |  |  |  |  |  |  |  |  |
| Minor shock |  |  |  |  | -0.0326** |  |  | -0.0318** | -0.0337** |
|  |  |  |  |  | (0.0152) |  |  | (0.0154) | (0.0155) |
| Major shock |  |  |  |  | -0.0307* |  |  | -0.0319* | -0.0327* |
|  |  |  |  |  | (0.0168) |  |  | (0.0171) | (0.0173) |
| **Inc. shock (ref: none)** |  |  |  |  |  |  |  |  |  |
| Minor shock |  |  |  |  |  | 0.0111 |  |  | 0.0238* |
|  |  |  |  |  |  | (0.0105) |  |  | (0.0136) |
| Major shock |  |  |  |  |  | 0.00808 |  |  | 0.0225 |
|  |  |  |  |  |  | (0.0118) |  |  | (0.0154) |
| **Emp.shock (ref:none)** |  |  |  |  |  |  |  |  |  |
| Temp. shock |  |  |  |  |  |  | -0.00573 | -0.00225 | -0.0184 |
|  |  |  |  |  |  |  | (0.00951) | (0.00969) | (0.0132) |
| Perm. shock |  |  |  |  |  |  | 0.00429 | 0.0111 | -0.00645 |
|  |  |  |  |  |  |  | (0.0197) | (0.0200) | (0.0227) |
|  |  |  |  |  |  |  |  |  |  |
| Constant | 0.546*** | 0.560*** | 0.470*** | 0.450*** | 0.456*** | 0.443*** | 0.453*** | 0.457*** | 0.454*** |
|  | (0.00658) | (0.00835) | (0.0126) | (0.0152) | (0.0155) | (0.0163) | (0.0167) | (0.0168) | (0.0169) |
|  |  |  |  |  |  |  |  |  |  |
| Observations | 4,598 | 4,583 | 4,539 | 4,114 | 4,050 | 4,059 | 4,055 | 4,003 | 3,982 |
| R-squared | 0.004 | 0.009 | 0.028 | 0.036 | 0.038 | 0.036 | 0.036 | 0.038 | 0.039 |

Note: Correlates of health IA in Italy, Germany, and the UK. Standard errors in parentheses *** p<0.01, ** p<0.05, * p<0.1

**Table B2: Health IA – UK 2016**

|  | (1) | (2) | (3) | (4) | (5) | (6) |
| --- | --- | --- | --- | --- | --- | --- |
| Age (reference: 25-64) |  |  |  |  |  |  |
| <25 | -0.0150 | -0.00199 | -0.0206 | -0.0225 | -0.0205 | -0.0217 |
|  | (0.0212) | (0.0214) | (0.0235) | (0.0237) | (0.0236) | (0.0238) |
| 65+ | -0.0143 | -0.0223 | -0.0227 | -0.0221 | -0.0220 | -0.0205 |
|  | (0.0152) | (0.0152) | (0.0165) | (0.0166) | (0.0167) | (0.0167) |
| Gender (reference: female) |  |  |  |  |  |  |
| Male | 0.0290** | 0.0424*** | 0.0388*** | 0.0379*** | 0.0378*** | 0.0372*** |
|  | (0.0122) | (0.0124) | (0.0131) | (0.0132) | (0.0131) | (0.0132) |
| Education level (reference: medium) |  |  |  |  |  |  |
| low education |  |  | 0.106** | 0.107** | 0.104** | 0.107** |
|  |  |  | (0.0413) | (0.0418) | (0.0417) | (0.0418) |
| high education |  |  | 0.0443*** | 0.0457*** | 0.0456*** | 0.0465*** |
|  |  |  | (0.0135) | (0.0135) | (0.0135) | (0.0135) |
| Income level (reference: medium) |  |  |  |  |  |  |
| low income |  |  | -0.0124 | -0.0136 | -0.0139 | -0.0156 |
|  |  |  | (0.0174) | (0.0175) | (0.0175) | (0.0176) |
| high income |  |  | -0.0430*** | -0.0435*** | -0.0448*** | -0.0444*** |
|  |  |  | (0.0154) | (0.0154) | (0.0154) | (0.0155) |
| Risk aversion (0-1 scale) |  |  |  |  |  |  |
|  |  | 0.129*** | 0.126*** | 0.124*** | 0.128*** | 0.127*** |
|  |  | (0.0231) | (0.0244) | (0.0246) | (0.0246) | (0.0248) |
| Health shock (reference: none) |  |  |  |  |  |  |
| Minor health shock |  |  |  | -0.0134 |  | -0.0146 |
|  |  |  |  | (0.0177) |  | (0.0182) |
| Major health shock |  |  |  | -0.0172 |  | -0.0167 |
|  |  |  |  | (0.0224) |  | (0.0230) |
| Income shock (reference: none) |  |  |  |  |  |  |
| Minor income shock |  |  |  |  | -0.00805 | -0.00233 |
|  |  |  |  |  | (0.0205) | (0.0210) |
| Major income shock |  |  |  |  | 0.0140 | 0.0199 |
|  |  |  |  |  | (0.0278) | (0.0284) |
|  |  |  |  |  |  |  |
| Constant | 0.441*** | 0.365*** | 0.357*** | 0.362*** | 0.357*** | 0.361*** |
|  | (0.00889) | (0.0161) | (0.0194) | (0.0201) | (0.0202) | (0.0206) |
|  |  |  |  |  |  |  |
| Observations | 1,951 | 1,934 | 1,746 | 1,738 | 1,739 | 1,734 |
| R-squared | 0.003 | 0.019 | 0.027 | 0.028 | 0.028 | 0.029 |

Note: Correlates of health IA in Italy, Germany, and the UK. Standard errors in parentheses *** p<0.01, ** p<0.05, * p<0.1

**Table B3: Income IA – 2020**

|  | (1) | (2) | (3) | (4) | (5) | (6) | (7) | (8) | (9) |
| --- | --- | --- | --- | --- | --- | --- | --- | --- | --- |
| **Age group (reference: 25-64)** |  |  |  |  |  |  |  |  |  |
| <25 |  | -0.0495*** | -0.0312** | -0.0360*** | -0.0259* | -0.0336** | -0.0308** | -0.0243* | -0.0237* |
|  |  | (0.0128) | (0.0128) | (0.0137) | (0.0141) | (0.0140) | (0.0140) | (0.0142) | (0.0143) |
| 65+ |  | 0.0372*** | 0.0219* | 0.0170 | 0.0173 | 0.0161 | 0.0146 | 0.0163 | 0.0154 |
|  |  | (0.0132) | (0.0131) | (0.0138) | (0.0139) | (0.0140) | (0.0141) | (0.0142) | (0.0142) |
| **Gender (reference: female)** |  |  |  |  |  |  |  |  |  |
| Male |  | -0.0137 | 0.000188 | 0.00143 | 0.00243 | 0.00203 | 0.00238 | 0.00330 | 0.00270 |
|  |  | (0.00860) | (0.00856) | (0.00902) | (0.00909) | (0.00911) | (0.00911) | (0.00917) | (0.00921) |
| **Education level (reference: medium)** |  |  |  |  |  |  |  |  |  |
| low education |  |  |  | -0.0201 | -0.0223 | -0.0221 | -0.0198 | -0.0229 | -0.0235 |
|  |  |  |  | (0.0161) | (0.0163) | (0.0163) | (0.0162) | (0.0164) | (0.0165) |
| high education |  |  |  | 0.0305*** | 0.0330*** | 0.0315*** | 0.0304*** | 0.0330*** | 0.0327*** |
|  |  |  |  | (0.00987) | (0.00994) | (0.00995) | (0.00994) | (0.01000) | (0.0100) |
| **Income level (reference: medium)** |  |  |  |  |  |  |  |  |  |
| low income |  |  |  | 0.0205** | 0.0196* | 0.0206* | 0.0211** | 0.0202* | 0.0196* |
|  |  |  |  | (0.0104) | (0.0105) | (0.0106) | (0.0105) | (0.0106) | (0.0107) |
| high income |  |  |  | -0.0341*** | -0.0346*** | -0.0338*** | -0.0363*** | -0.0357*** | -0.0347*** |
|  |  |  |  | (0.0121) | (0.0122) | (0.0122) | (0.0122) | (0.0122) | (0.0123) |
| **Risk aversion (0-1 scale)** |  |  |  |  |  |  |  |  |  |
|  |  |  | 0.211*** | 0.218*** | 0.212*** | 0.216*** | 0.213*** | 0.210*** | 0.210*** |
|  |  |  | (0.0168) | (0.0177) | (0.0178) | (0.0179) | (0.0179) | (0.0180) | (0.0181) |
| **Country (reference: UK)** |  |  |  |  |  |  |  |  |  |
| Italy | -0.0151 | -0.0135 | -0.00770 | 0.00403 | 0.00554 | 0.00442 | 0.00608 | 0.00599 | 0.00467 |
|  | (0.00963) | (0.00969) | (0.00958) | (0.0102) | (0.0103) | (0.0103) | (0.0103) | (0.0104) | (0.0104) |
| Germany | 0.0543*** | 0.0517*** | 0.0452*** | 0.0453*** | 0.0461*** | 0.0471*** | 0.0467*** | 0.0475*** | 0.0486*** |
|  | (0.0117) | (0.0117) | (0.0116) | (0.0125) | (0.0126) | (0.0126) | (0.0126) | (0.0127) | (0.0127) |
| **Health shock (reference: none)** |  |  |  |  |  |  |  |  |  |
| Minor health shock |  |  |  |  | -0.0246 |  |  | -0.0230 | -0.0237 |
|  |  |  |  |  | (0.0154) |  |  | (0.0156) | (0.0157) |
| Major health shock |  |  |  |  | -0.0463*** |  |  | -0.0443** | -0.0437** |
|  |  |  |  |  | (0.0172) |  |  | (0.0176) | (0.0178) |
| **Income shock (reference: none)** |  |  |  |  |  |  |  |  |  |
| Minor income shock |  |  |  |  |  | 0.00898 |  |  | 0.0217 |
|  |  |  |  |  |  | (0.0106) |  |  | (0.0136) |
| Major income shock |  |  |  |  |  | 8.04e-05 |  |  | 0.0218 |
|  |  |  |  |  |  | (0.0120) |  |  | (0.0155) |
| **Employment shock (reference: none)** |  |  |  |  |  |  |  |  |  |
| Temporary employment shock |  |  |  |  |  |  | -0.00577 | -0.000393 | -0.0157 |
|  |  |  |  |  |  |  | (0.00956) | (0.00973) | (0.0132) |
| Permanent employment shock |  |  |  |  |  |  | -0.0374* | -0.0278 | -0.0417* |
|  |  |  |  |  |  |  | (0.0200) | (0.0203) | (0.0229) |
|  |  |  |  |  |  |  |  |  |  |
| Constant | 0.579*** | 0.588*** | 0.466*** | 0.450*** | 0.456*** | 0.448*** | 0.456*** | 0.458*** | 0.457*** |
|  | (0.00671) | (0.00851) | (0.0128) | (0.0153) | (0.0156) | (0.0164) | (0.0168) | (0.0169) | (0.0170) |
|  |  |  |  |  |  |  |  |  |  |
| Observations | 4,811 | 4,796 | 4,751 | 4,296 | 4,234 | 4,238 | 4,237 | 4,186 | 4,163 |
| R-squared | 0.007 | 0.013 | 0.045 | 0.053 | 0.054 | 0.052 | 0.053 | 0.055 | 0.055 |

Note: Correlates of income-IA in Italy, Germany, and the UK. Standard errors in parentheses *** p<0.01, ** p<0.05, * p<0.1

**Table B4: Income IA – UK 2016**

|  | (1) | (2) | (3) | (4) | (5) | (6) |
| --- | --- | --- | --- | --- | --- | --- |
|  | Income IA | Income IA | Income IA | Income IA | Income IA | Income IA |
| **Age group (reference: 25-64)** |  |  |  |  |  |  |
| <25 | -0.00142 | 0.0202 | 0.000734 | 0.000788 | 0.00266 | 0.00330 |
|  | (0.0215) | (0.0216) | (0.0236) | (0.0238) | (0.0237) | (0.0239) |
| 65+ | -0.0178 | -0.0298** | -0.0292* | -0.0293* | -0.0292* | -0.0276* |
|  | (0.0153) | (0.0152) | (0.0164) | (0.0165) | (0.0166) | (0.0167) |
| **Gender (reference: female)** |  |  |  |  |  |  |
| Male | 0.00474 | 0.0249** | 0.0251* | 0.0240* | 0.0241* | 0.0235* |
|  | (0.0123) | (0.0124) | (0.0131) | (0.0131) | (0.0131) | (0.0131) |
| **Education level (reference: medium)** |  |  |  |  |  |  |
| low education |  |  | 0.0933** | 0.0953** | 0.0914** | 0.0944** |
|  |  |  | (0.0408) | (0.0412) | (0.0412) | (0.0413) |
| high education |  |  | 0.0437*** | 0.0454*** | 0.0441*** | 0.0453*** |
|  |  |  | (0.0134) | (0.0135) | (0.0135) | (0.0135) |
| **Income level (reference: medium)** |  |  |  |  |  |  |
| low income |  |  | 0.0191 | 0.0185 | 0.0177 | 0.0166 |
|  |  |  | (0.0174) | (0.0175) | (0.0175) | (0.0176) |
| high income |  |  | -0.0547*** | -0.0562*** | -0.0564*** | -0.0566*** |
|  |  |  | (0.0153) | (0.0154) | (0.0154) | (0.0154) |
| **Risk aversion (0-1 scale)** |  |  |  |  |  |  |
|  |  | 0.184*** | 0.188*** | 0.183*** | 0.188*** | 0.185*** |
|  |  | (0.0231) | (0.0244) | (0.0246) | (0.0247) | (0.0248) |
| **Health shock (reference: none)** |  |  |  |  |  |  |
| Minor health shock |  |  |  | -0.0296* |  | -0.0309* |
|  |  |  |  | (0.0178) |  | (0.0182) |
| Major health shock |  |  |  | -0.0393* |  | -0.0419* |
|  |  |  |  | (0.0223) |  | (0.0229) |
| **Income shock (reference: none)** |  |  |  |  |  |  |
| Minor income shock |  |  |  |  | -0.0123 | -0.00259 |
|  |  |  |  |  | (0.0204) | (0.0209) |
| Major income shock |  |  |  |  | 0.00783 | 0.0207 |
|  |  |  |  |  | (0.0277) | (0.0283) |
|  |  |  |  |  |  |  |
| Constant | 0.530*** | 0.421*** | 0.407*** | 0.418*** | 0.409*** | 0.418*** |
|  | (0.00898) | (0.0162) | (0.0194) | (0.0201) | (0.0202) | (0.0206) |
|  |  |  |  |  |  |  |
| Observations | 1,943 | 1,927 | 1,743 | 1,735 | 1,735 | 1,730 |
| R-squared | 0.001 | 0.033 | 0.047 | 0.050 | 0.047 | 0.050 |
|  | (1) | (2) | (3) | (4) | (5) | (6) |

Note: Correlates of income-IA in Italy, Germany, and the UK. Standard errors in parentheses *** p<0.01, ** p<0.05, * p<0.1

# Appendix C: DiD regressions

Tables C1 to C8 all refer to DiD analysis carried out the UK samples in 2016 and 2020 to provide additional supporting detail for the estimates summarised Table 4 and to report on alternative variants of the model. Tables C1 to C4 report the DID estimates for $\gamma^{h}$ (IA in the health dimension) and Tables C5 to C8 report the DID estimates for $\gamma^{y}$ (IA in the income dimension).

Tables C1 and C5 report the estimates for the simplest variants of the DiD model. These variants are without conditioning covariates and with single-source vulnerability. They also set either the shock indicator $s_{it}$ or the vulnerability indicator $v_{it}$ to be inactive (see equation 2).

Tables C2 and C6 report the DiD estimates with multi-source vulnerability, Tables C3 and C7 report simple DiD with single-source vulnerability and conditioning covariates, and finally Tables C4 and C8 rep ort DiD with multi-source vulnerability and conditioning covariates.

**Table C1:** $\gamma^{h}$ **DiD without conditioning covariates, single-source vulnerability**

| (1) (2) | | (3) | (4) | | (5) | (6) |
| --- | --- | --- | --- | --- | --- | --- |
| Year = 2020 | | 0.100*** | 0.0932*** | | 0.0995*** | 0.100*** |
|  | (0.0113) | (0.00983) | (0.00932) | (0.0100) | 0.0931*** | 0.0849*** |
| High regional risk | 0.0166 |  |  |  | (0.00958) | (0.0110) |
|  | (0.0130) |  |  |  |  |  |
| 2020 x High regional risk | -0.0122 |  |  |  |  |  |
|  | (0.0185) |  |  |  |  |  |
| High age-based risk |  | -0.00904 |  |  |  |  |
|  |  | (0.0155) |  |  |  |  |
| 2020 x High age-based risk |  | 0.0161 |  |  |  |  |
|  |  | (0.0239) |  |  |  |  |
| Serious health shock |  |  | -0.0227 |  |  |  |
|  |  |  | (0.0216) |  |  |  |
| 2020 x Serious health shock |  |  | -0.0527 |  |  |  |
|  |  |  | (0.0326) |  |  |  |
| Minor or serious health shock |  |  |  | -0.0217 |  |  |
|  |  |  |  | (0.0145) |  |  |
| 2020 x Minor or serious health shock |  |  |  | -0.0372* |  |  |
|  |  |  |  | (0.0224) |  |  |
| Serious income shock |  |  |  |  |  |  |
|  |  |  |  |  | 0.0131 |  |
| 2020 x Serious income shock |  |  |  |  | (0.0275) |  |
|  |  |  |  |  | 0.00577 |  |
| Minor or serious income shock |  |  |  |  | (0.0319) |  |
|  |  |  |  |  |  | -0.000562 |
| 2020 x Minor or serious income shock |  |  |  |  |  | (0.0168) |
|  |  |  |  |  |  | 0.0248 |
| Constant | 0.444*** | 0.452*** | 0.452*** | 0.455*** |  | (0.0211) |
|  | (0.00787) | (0.00704) | (0.00658) | (0.00721) | 0.449*** | 0.450*** |
| Observations | 3,846 | 3,846 | 3,846 | 3,846 | (0.00645) | (0.00687) |
| R-squared | 0.030 | 0.029 | 0.032 | 0.033 | 3,846 | 3,846 |
|  |  |  |  |  |  |  |

Note: Standard errors in parentheses. *** p<0.01, ** p<0.05, * p<0.1

**Table C2:** $\gamma^{h}$ **DiD without conditioning covariates, multiple-source vulnerability**

|  | (1) | (2) | (3) | (4) | (5) | (6) | (7) | (8) |
| --- | --- | --- | --- | --- | --- | --- | --- | --- |
| Year = 2020 | 0.0993*** | 0.0936*** | 0.0941*** | 0.0971*** | 0.0943*** | 0.0996*** | 0.0955*** | 0.0952*** |
|  | (0.00918) | (0.00898) | (0.00896) | (0.00908) | (0.00919) | (0.00902) | (0.00895) | (0.00894) |
| High regional and age risk | 0.0581** |  |  |  |  |  |  |  |
|  | (0.0262) |  |  |  |  |  |  |  |
| 2020 * High regional and age risk | -0.0516 |  |  |  |  |  |  |  |
|  | (0.0395) |  |  |  |  |  |  |  |
| Health shock and high age risk |  | -0.0474 |  |  |  |  |  |  |
|  |  | (0.0472) |  |  |  |  |  |  |
| 2020 * Health shock and high age risk |  | 0.239*** |  |  |  |  |  |  |
|  |  | (0.0840) |  |  |  |  |  |  |
| Income shock and high age risk |  |  | -0.0796 |  |  |  |  |  |
|  |  |  | (0.113) |  |  |  |  |  |
| 2020 * Income shock and high age risk |  |  | 0.234* |  |  |  |  |  |
|  |  |  | (0.128) |  |  |  |  |  |
| Health shock and high regional risk |  |  |  | -0.0518 |  |  |  |  |
|  |  |  |  | (0.0344) |  |  |  |  |
| 2020 * Health shock and high regional risk |  |  |  | -0.0329 |  |  |  |  |
|  |  |  |  | (0.0494) |  |  |  |  |
| Income shock and high regional risk |  |  |  |  | -0.0192 |  |  |  |
|  |  |  |  |  | (0.0437) |  |  |  |
| 2020 * Income shock and high regional risk |  |  |  |  | 0.0351 |  |  |  |
|  |  |  |  |  | (0.0492) |  |  |  |
| Income shock and health shock |  |  |  |  |  | -0.0841* |  |  |
|  |  |  |  |  |  | (0.0485) |  |  |
| 2020 * Income shock and health shock |  |  |  |  |  | -0.0695 |  |  |
|  |  |  |  |  |  | (0.0604) |  |  |
| Health shock and high regional and age risk |  |  |  |  |  |  | 0.0366 |  |
|  |  |  |  |  |  |  | (0.0981) |  |
| 2020 * Health shock and high regional and age risk |  |  |  |  |  |  | 0.141 |  |
|  |  |  |  |  |  |  | (0.139) |  |
| Income shock and high regional and age risk |  |  |  |  |  |  |  | 0.106 |
|  |  |  |  |  |  |  |  | (0.196) |
| 2020 * Income shock and high regional and age risk |  |  |  |  |  |  |  | 0.159 |
|  |  |  |  |  |  |  |  | (0.222) |
| Constant | 0.446*** | 0.451*** | 0.450*** | 0.451*** | 0.450*** | 0.451*** | 0.450*** | 0.450*** |
|  | (0.00647) | (0.00632) | (0.00628) | (0.00638) | (0.00634) | (0.00631) | (0.00628) | (0.00627) |
| Observations | 3,846 | 3,846 | 3,846 | 3,846 | 3,846 | 3,846 | 3,846 | 3,846 |
| R-squared | 0.030 | 0.031 | 0.031 | 0.031 | 0.029 | 0.035 | 0.030 | 0.031 |
|  |  |  |  |  |  |  |  |  |

Note: Standard errors in parentheses. *** p<0.01, ** p<0.05, * p<0.1

**Table C3:** $\gamma^{h}$ **DiD with conditioning covariates,** single-source vulnerability

|  | (1) | (2) | (3) | (4) | (5) | (6) |
| --- | --- | --- | --- | --- | --- | --- |
| **Age group (reference: 25-64)** |  |  |  |  |  |  |
| <25 | -0.0395*** | -0.0395*** | -0.0354** | -0.0351** | -0.0406*** | -0.0417*** |
|  | (0.0144) | (0.0145) | (0.0145) | (0.0145) | (0.0145) | (0.0144) |
| 65+ | -0.0156 | -0.0194 | -0.0163 | -0.0164 | -0.0151 | -0.0145 |
|  | (0.0128) | (0.0167) | (0.0128) | (0.0128) | (0.0128) | (0.0129) |
| **Gender (reference: female)** |  |  |  |  |  |  |
| Male | -0.000202 | 0.000153 | 0.000491 | 0.000847 | -0.000305 | 5.82e-05 |
|  | (0.00949) | (0.00951) | (0.00949) | (0.00950) | (0.00951) | (0.00949) |
| **Education level (reference: medium)** |  |  |  |  |  |  |
| low education | -0.00690 | -0.00673 | -0.00526 | -0.00298 | -0.00473 | -0.00483 |
|  | (0.0240) | (0.0241) | (0.0240) | (0.0240) | (0.0240) | (0.0240) |
| high education | 0.0586*** | 0.0588*** | 0.0583*** | 0.0589*** | 0.0589*** | 0.0595*** |
|  | (0.00978) | (0.00979) | (0.00978) | (0.00978) | (0.00979) | (0.00979) |
| **Income level (reference: medium)** |  |  |  |  |  |  |
| low income | -0.00445 | -0.00413 | -0.00497 | -0.00423 | -0.00428 | -0.00283 |
|  | (0.0119) | (0.0119) | (0.0119) | (0.0119) | (0.0119) | (0.0119) |
| high income | -0.0399*** | -0.0396*** | -0.0393*** | -0.0389*** | -0.0389*** | -0.0387*** |
|  | (0.0114) | (0.0114) | (0.0114) | (0.0114) | (0.0114) | (0.0114) |
| **Risk attitude (reference: moderate)** |  |  |  |  |  |  |
| extremely risk loving | 0.125*** | 0.124*** | 0.122*** | 0.120*** | 0.125*** | 0.126*** |
|  | (0.0182) | (0.0182) | (0.0182) | (0.0183) | (0.0183) | (0.0183) |
| extremely risk averse |  |  |  |  |  |  |
|  | 0.108*** | 0.0993*** | 0.103*** | 0.104*** | 0.0976*** | 0.0862*** |
|  | (0.0119) | (0.0103) | (0.00983) | (0.0105) | (0.0101) | (0.0116) |
|  |  |  |  |  |  |  |
| Year = 2020 | 0.0227* |  |  |  |  |  |
|  | (0.0137) |  |  |  |  |  |
| High regional risk | -0.0210 |  |  |  |  |  |
|  | (0.0192) |  |  |  |  |  |
| 2020 * High regional risk |  | - |  |  |  |  |
|  |  |  |  |  |  |  |
| 2020 * High age-based risk (65+) |  | 0.00640 |  |  |  |  |
|  |  | (0.0252) |  |  |  |  |
| Serious health shock |  |  | -0.0140 |  |  |  |
|  |  |  | (0.0229) |  |  |  |
| 2020 * Serious health shock |  |  | -0.0477 |  |  |  |
|  |  |  | (0.0340) |  |  |  |
| Minor or serious health shock |  |  |  | -0.0153 |  |  |
|  |  |  |  | (0.0152) |  |  |
| 2020 * Minor or serious health shock |  |  |  | -0.0327 |  |  |
|  |  |  |  | (0.0233) |  |  |
| Serious income shock |  |  |  |  | 0.0150 |  |
|  |  |  |  |  | (0.0284) |  |
| 2020 * Serious income shock |  |  |  |  | 0.00427 |  |
|  |  |  |  |  | (0.0328) |  |
| Minor or serious income shock |  |  |  |  |  | -0.000862 |
|  |  |  |  |  |  | (0.0177) |
| 2020 * Minor or serious income shock |  |  |  |  |  | 0.0314 |
|  |  |  |  |  |  | (0.0220) |
| Constant | 0.362*** | 0.371*** | 0.372*** | 0.375*** | 0.368*** | 0.368*** |
|  | (0.0160) | (0.0153) | (0.0153) | (0.0158) | (0.0154) | (0.0157) |
|  |  |  |  |  |  |  |
| Observations | 3,494 | 3,494 | 3,494 | 3,494 | 3,494 | 3,494 |
| R-squared | 0.059 | 0.058 | 0.060 | 0.060 | 0.058 | 0.059 |
|  |  |  |  |  |  |  |

Note: Standard errors in parentheses. *** p<0.01, ** p<0.05, * p<0.1

**Table C4:** $\gamma^{h}$ **DiD with conditioning covariates, multiple-source vulnerability**

|  | (1) | (2) | (3) | (4) | (5) | (6) | (7) | (8) |
| --- | --- | --- | --- | --- | --- | --- | --- | --- |
| **Age group (reference: 25-64)** |  |  |  |  |  |  |  |  |
| <25 | -0.0403*** | -0.0392*** | -0.0392*** | -0.0365** | -0.0398*** | -0.0333** | -0.0395*** | -0.0396*** |
|  | (0.0144) | (0.0144) | (0.0144) | (0.0145) | (0.0145) | (0.0145) | (0.0144) | (0.0144) |
| 65+ | -0.0317** | -0.0202 | -0.0204 | -0.0165 | -0.0165 | -0.0185 | -0.0194 | -0.0185 |
|  | (0.0149) | (0.0132) | (0.0130) | (0.0128) | (0.0128) | (0.0128) | (0.0129) | (0.0128) |
| **Gender (reference: female)** |  |  |  |  |  |  |  |  |
| Male | -0.000495 | 0.000183 | -0.000341 | 0.000352 | -2.20e-05 | 0.000805 | 9.21e-05 | 9.00e-05 |
|  | (0.00949) | (0.00949) | (0.00949) | (0.00949) | (0.00951) | (0.00947) | (0.00949) | (0.00949) |
| **Education level (reference: medium)** |  |  |  |  |  |  |  |  |
| low education | -0.00582 | -0.00855 | -0.00703 | -0.00407 | -0.00604 | -0.00603 | -0.00956 | -0.00565 |
|  | (0.0241) | (0.0240) | (0.0240) | (0.0241) | (0.0240) | (0.0240) | (0.0241) | (0.0240) |
| high education | 0.0592*** | 0.0591*** | 0.0589*** | 0.0587*** | 0.0587*** | 0.0575*** | 0.0588*** | 0.0585*** |
|  | (0.00978) | (0.00978) | (0.00978) | (0.00978) | (0.00979) | (0.00977) | (0.00978) | (0.00979) |
| **Income level (reference: medium)** |  |  |  |  |  |  |  |  |
| low income | -0.00362 | -0.00363 | -0.00422 | -0.00491 | -0.00397 | -0.00427 | -0.00279 | -0.00449 |
|  | (0.0119) | (0.0119) | (0.0119) | (0.0119) | (0.0120) | (0.0119) | (0.0119) | (0.0119) |
| high income | -0.0393*** | -0.0397*** | -0.0399*** | -0.0391*** | -0.0394*** | -0.0402*** | -0.0392*** | -0.0398*** |
|  | (0.0113) | (0.0113) | (0.0114) | (0.0114) | (0.0114) | (0.0113) | (0.0114) | (0.0114) |
| **Risk attitude (reference: moderate)** |  |  |  |  |  |  |  |  |
| extremely risk loving | 0.124*** | 0.123*** | 0.125*** | 0.123*** | 0.124*** | 0.119*** | 0.123*** | 0.124*** |
|  | (0.0182) | (0.0182) | (0.0182) | (0.0182) | (0.0183) | (0.0182) | (0.0182) | (0.0182) |
| extremely risk averse |  |  |  |  |  |  |  |  |
|  | 0.104*** | 0.0981*** | 0.0984*** | 0.102*** | 0.1000*** | 0.103*** | 0.1000*** | 0.0998*** |
|  | (0.00970) | (0.00950) | (0.00948) | (0.00962) | (0.00972) | (0.00954) | (0.00947) | (0.00946) |
|  |  |  |  |  |  |  |  |  |
| Year = 2020 | 0.104*** | 0.0981*** | 0.0984*** | 0.102*** | 0.1000*** | 0.103*** | 0.1000*** | 0.0998*** |
|  | (0.00970) | (0.00950) | (0.00948) | (0.00962) | (0.00972) | (0.00954) | (0.00947) | (0.00946) |
| High regional and age risk | 0.0833*** |  |  |  |  |  |  |  |
|  | (0.0306) |  |  |  |  |  |  |  |
| 2020 * High regional and age risk | -0.0762* |  |  |  |  |  |  |  |
|  | (0.0413) |  |  |  |  |  |  |  |
| Health shock and high age risk |  | -0.0234 |  |  |  |  |  |  |
|  |  | (0.0523) |  |  |  |  |  |  |
| 2020 * Health shock and high age risk |  | 0.206** |  |  |  |  |  |  |
|  |  | (0.0876) |  |  |  |  |  |  |
| Income shock and high age risk |  |  | -0.0966 |  |  |  |  |  |
|  |  |  | (0.123) |  |  |  |  |  |
| 2020 * Income shock and high age risk |  |  | 0.249* |  |  |  |  |  |
|  |  |  | (0.140) |  |  |  |  |  |
| Health shock and high regional risk |  |  |  | -0.0230 |  |  |  |  |
|  |  |  |  | (0.0361) |  |  |  |  |
| 2020 * Health shock and high regional risk |  |  |  | -0.0531 |  |  |  |  |
|  |  |  |  | (0.0510) |  |  |  |  |
| Income shock and high regional risk |  |  |  |  | 0.000815 |  |  |  |
|  |  |  |  |  | (0.0441) |  |  |  |
| 2020 * Income shock and high regional risk |  |  |  |  | 0.00343 |  |  |  |
|  |  |  |  |  | (0.0498) |  |  |  |
| Income shock and health shock |  |  |  |  |  | -0.103** |  |  |
|  |  |  |  |  |  | (0.0523) |  |  |
| 2020 * Income shock and health shock |  |  |  |  |  | -0.0371 |  |  |
|  |  |  |  |  |  | (0.0638) |  |  |
| Health shock and high regional and age risk |  |  |  |  |  |  | 0.124 |  |
|  |  |  |  |  |  |  | (0.113) |  |
| 2020 * Health shock and high regional and age risk |  |  |  |  |  |  | 0.0144 |  |
|  |  |  |  |  |  |  | (0.153) |  |
| Income shock and high regional and age risk |  |  |  |  |  |  |  | 0.139 |
|  |  |  |  |  |  |  |  | (0.274) |
| 2020 * Income shock and high regional and age risk |  |  |  |  |  |  |  | 0.0444 |
|  |  |  |  |  |  |  |  | (0.300) |
| Constant | 0.368*** | 0.371*** | 0.371*** | 0.371*** | 0.370*** | 0.375*** | 0.370*** | 0.371*** |
|  | (0.0152) | (0.0151) | (0.0151) | (0.0152) | (0.0152) | (0.0151) | (0.0151) | (0.0151) |
|  |  |  |  |  |  |  |  |  |
| Observations | 3,494 | 3,494 | 3,494 | 3,494 | 3,494 | 3,494 | 3,494 | 3,494 |
| R-squared | 0.060 | 0.060 | 0.060 | 0.059 | 0.058 | 0.063 | 0.059 | 0.059 |

Note: Standard errors in parentheses. *** p<0.01, ** p<0.05, * p<0.1

**Table C5:** $\gamma^{y}$ **DiD without conditioning covariates, single-source vulnerability**

|  | (1) | (2) | (3) | (4) | (5) | (6) |
| --- | --- | --- | --- | --- | --- | --- |
| Year = 2020 | 0.0458*** | 0.0458*** | 0.0522*** | 0.0521*** | 0.0512*** | 0.0444*** |
|  | (0.0114) | (0.00998) | (0.00943) | (0.0101) | (0.00969) | (0.0112) |
| High regional risk | -0.000264 |  |  |  |  |  |
|  | (0.0134) |  |  |  |  |  |
| 2020 * High regional risk | 0.0136 |  |  |  |  |  |
|  | (0.0187) |  |  |  |  |  |
| High age based risk |  | -0.0171 |  |  |  |  |
|  |  | (0.0159) |  |  |  |  |
| 2020 * High age based risk |  | 0.0279 |  |  |  |  |
|  |  | (0.0240) |  |  |  |  |
| Serious health shock |  |  | -0.0355 |  |  |  |
|  |  |  | (0.0220) |  |  |  |
| 2020 * Serious health shock |  |  | -0.0325 |  |  |  |
|  |  |  | (0.0335) |  |  |  |
| Minor or serious health shock |  |  |  | -0.0332** |  |  |
|  |  |  |  | (0.0149) |  |  |
| 2020 * Minor or serious health shock |  |  |  | -0.0262 |  |  |
|  |  |  |  | (0.0229) |  |  |
| Serious income shock |  |  |  |  | 0.00475 |  |
|  |  |  |  |  | (0.0282) |  |
| 2020 * Serious income shock |  |  |  |  | -0.00462 |  |
|  |  |  |  |  | (0.0325) |  |
| Minor or serious income shock |  |  |  |  |  | -0.00442 |
|  |  |  |  |  |  | (0.0172) |
| 2020 * Minor or serious income shock |  |  |  |  |  | 0.0171 |
|  |  |  |  |  |  | (0.0214) |
| Constant | 0.528*** | 0.532*** | 0.532*** | 0.536*** | 0.528*** | 0.529*** |
|  | (0.00806) | (0.00721) | (0.00674) | (0.00739) | (0.00661) | (0.00704) |
|  |  |  |  |  |  |  |
| Observations | 3,916 | 3,916 | 3,916 | 3,916 | 3,916 | 3,916 |
| R-squared | 0.008 | 0.008 | 0.011 | 0.012 | 0.008 | 0.008 |
|  |  |  |  |  |  |  |

Note: Standard errors in parentheses. *** p<0.01, ** p<0.05, * p<0.1

**Table C6:** $\gamma^{y}$ **DiD without conditioning covariates, multiple-source vulnerability**

|  | (1) | (2) | (3) | (4) | (5) | (6) | (7) | (8) |
| --- | --- | --- | --- | --- | --- | --- | --- | --- |
|  | $\gamma^{y}$ | $\gamma^{y}$ | $\gamma^{y}$ | $\gamma^{y}$ | $\gamma^{y}$ | $\gamma^{y}$ | $\gamma^{y}$ | $\gamma^{y}$ |
| Year = 2020 | 0.0508*** | 0.0484*** | 0.0505*** | 0.0505*** | 0.0499*** | 0.0533*** | 0.0499*** | 0.0508*** |
|  | (0.00931) | (0.00911) | (0.00909) | (0.00920) | (0.00931) | (0.00915) | (0.00907) | (0.00906) |
| High regional and age risk | 0.0162 |  |  |  |  |  |  |  |
|  | (0.0268) |  |  |  |  |  |  |  |
| 2020 * High regional and age risk | 0.00718 |  |  |  |  |  |  |  |
|  | (0.0398) |  |  |  |  |  |  |  |
| Health shock and high age risk |  | -0.0765* |  |  |  |  |  |  |
|  |  | (0.0464) |  |  |  |  |  |  |
| 2020 * Health shock and high age risk |  | 0.200** |  |  |  |  |  |  |
|  |  | (0.0848) |  |  |  |  |  |  |
| Income shock and high age risk |  |  | -0.0655 |  |  |  |  |  |
|  |  |  | (0.116) |  |  |  |  |  |
| 2020 * Income shock and high age risk |  |  | 0.0856 |  |  |  |  |  |
|  |  |  | (0.130) |  |  |  |  |  |
| Health shock and high regional risk |  |  |  | -0.0834** |  |  |  |  |
|  |  |  |  | (0.0349) |  |  |  |  |
| 2020 * Health shock and high regional risk |  |  |  | 0.00521 |  |  |  |  |
|  |  |  |  | (0.0501) |  |  |  |  |
| Income shock and high regional risk |  |  |  |  | -0.0579 |  |  |  |
|  |  |  |  |  | (0.0447) |  |  |  |
| 2020 * Income shock and high regional risk |  |  |  |  | 0.0554 |  |  |  |
|  |  |  |  |  | (0.0501) |  |  |  |
| Income shock and health shock |  |  |  |  |  | -0.0647 |  |  |
|  |  |  |  |  |  | (0.0497) |  |  |
| 2020 * Income shock and health shock |  |  |  |  |  | -0.0480 |  |  |
|  |  |  |  |  |  | (0.0618) |  |  |
| Health shock and high regional and age risk |  |  |  |  |  |  | -0.129 |  |
|  |  |  |  |  |  |  | (0.0898) |  |
| 2020 * Health shock and high regional and age risk |  |  |  |  |  |  | 0.217 |  |
|  |  |  |  |  |  |  | (0.135) |  |
| Income shock and high regional and age risk |  |  |  |  |  |  |  | -0.0839 |
|  |  |  |  |  |  |  |  | (0.200) |
| 2020 * Income shock and high regional and age risk |  |  |  |  |  |  |  | 0.0920 |
|  |  |  |  |  |  |  |  | (0.227) |
| Constant | 0.527*** | 0.530*** | 0.528*** | 0.531*** | 0.529*** | 0.529*** | 0.529*** | 0.528*** |
|  | (0.00663) | (0.00648) | (0.00643) | (0.00653) | (0.00649) | (0.00647) | (0.00644) | (0.00643) |
|  |  |  |  |  |  |  |  |  |
| Observations | 3,916 | 3,916 | 3,916 | 3,916 | 3,916 | 3,916 | 3,916 | 3,916 |
| R-squared | 0.008 | 0.009 | 0.008 | 0.011 | 0.008 | 0.011 | 0.009 | 0.008 |

Note: Standard errors in parentheses. *** p<0.01, ** p<0.05, * p<0.1

**Table C7** $\gamma^{y}$: **DiD with conditioning covariates, single-source vulnerability**

|  | (1) | (2) | (3) | (4) | (5) | (6) |
| --- | --- | --- | --- | --- | --- | --- |
| **Age group (reference: 25-64)** |  |  |  |  |  |  |
| <25 | -0.0304** | -0.0287* | -0.0264* | -0.0253* | -0.0295** | -0.0303** |
|  | (0.0147) | (0.0147) | (0.0147) | (0.0147) | (0.0147) | (0.0147) |
| 65+ | -0.0216* | -0.0272 | -0.0215* | -0.0216* | -0.0208 | -0.0202 |
|  | (0.0128) | (0.0170) | (0.0128) | (0.0127) | (0.0128) | (0.0129) |
| **Gender (reference: female)** |  |  |  |  |  |  |
| Male | -0.00885 | -0.00841 | -0.00860 | -0.00824 | -0.00874 | -0.00871 |
|  | (0.00952) | (0.00954) | (0.00952) | (0.00952) | (0.00953) | (0.00952) |
| **Education level (reference: medium)** |  |  |  |  |  |  |
| low education | 0.00116 | -0.000179 | 0.00282 | 0.00433 | 0.00156 | 0.00198 |
|  | (0.0242) | (0.0243) | (0.0242) | (0.0242) | (0.0242) | (0.0242) |
| high education | 0.0546*** | 0.0548*** | 0.0547*** | 0.0555*** | 0.0550*** | 0.0553*** |
|  | (0.00981) | (0.00981) | (0.00981) | (0.00980) | (0.00982) | (0.00981) |
| **Income level (reference: medium)** |  |  |  |  |  |  |
| low income | 0.0113 | 0.0105 | 0.00972 | 0.0103 | 0.0104 | 0.0115 |
|  | (0.0120) | (0.0120) | (0.0120) | (0.0120) | (0.0120) | (0.0120) |
| high income | -0.0443*** | -0.0447*** | -0.0446*** | -0.0443*** | -0.0441*** | -0.0439*** |
|  | (0.0114) | (0.0114) | (0.0114) | (0.0114) | (0.0114) | (0.0114) |
| **Risk attitude (reference: moderate)** |  |  |  |  |  |  |
| extremely risk loving | 0.195*** | 0.194*** | 0.192*** | 0.190*** | 0.196*** | 0.196*** |
|  | (0.0183) | (0.0183) | (0.0183) | (0.0184) | (0.0184) | (0.0184) |
| extremely risk averse |  |  |  |  |  |  |
|  | 0.0433*** | 0.0475*** | 0.0495*** | 0.0477*** | 0.0484*** | 0.0387*** |
|  | (0.0119) | (0.0104) | (0.00985) | (0.0106) | (0.0101) | (0.0116) |
|  |  |  |  |  |  |  |
| Year = 2020 | 0.00113 |  |  |  |  |  |
|  | (0.0139) |  |  |  |  |  |
| High regional risk | 0.0164 |  |  |  |  |  |
|  | (0.0193) |  |  |  |  |  |
| 2020 * High regional risk |  | - |  |  |  |  |
|  |  |  |  |  |  |  |
| 2020 * High age based risk (65+) |  | 0.0121 |  |  |  |  |
|  |  | (0.0251) |  |  |  |  |
| Serious health shock |  |  | -0.0324 |  |  |  |
|  |  |  | (0.0231) |  |  |  |
| 2020 * Serious health shock |  |  | -0.0113 |  |  |  |
|  |  |  | (0.0345) |  |  |  |
| Minor or serious health shock |  |  |  | -0.0323** |  |  |
|  |  |  |  | (0.0155) |  |  |
| 2020 * Minor or serious health shock |  |  |  | -0.00758 |  |  |
|  |  |  |  | (0.0236) |  |  |
| Serious income shock |  |  |  |  | 0.0133 |  |
|  |  |  |  |  | (0.0288) |  |
| 2020 * Serious income shock |  |  |  |  | -0.00316 |  |
|  |  |  |  |  | (0.0331) |  |
| Minor or serious income shock |  |  |  |  |  | -0.00223 |
|  |  |  |  |  |  | (0.0179) |
| 2020 * Minor or serious income shock |  |  |  |  |  | 0.0249 |
|  |  |  |  |  |  | (0.0221) |
| Constant | 0.416*** | 0.417*** | 0.420*** | 0.426*** | 0.414*** | 0.415*** |
|  | (0.0161) | (0.0154) | (0.0154) | (0.0158) | (0.0154) | (0.0158) |
|  |  |  |  |  |  |  |
| Observations | 3,569 | 3,569 | 3,569 | 3,569 | 3,569 | 3,569 |
| R-squared | 0.055 | 0.055 | 0.056 | 0.057 | 0.055 | 0.056 |
|  |  |  |  |  |  |  |

Note: Standard errors in parentheses. *** p<0.01, ** p<0.05, * p<0.1

**Table C8:** $\gamma^{y}$ **DiD with conditioning covariates, multiple-source vulnerability**

|  | (1) | (2) | (3) | (4) | (5) | (6) | (7) | (8) |
| --- | --- | --- | --- | --- | --- | --- | --- | --- |
| **Age group (reference: 25-64)** |  |  |  |  |  |  |  |  |
| <25 | -0.0291** | -0.0286* | -0.0290** | -0.0264* | -0.0292** | -0.0257* | -0.0290** | -0.0291** |
|  | (0.0146) | (0.0146) | (0.0146) | (0.0147) | (0.0147) | (0.0147) | (0.0146) | (0.0146) |
| 65+ | -0.0319** | -0.0217* | -0.0217* | -0.0221* | -0.0221* | -0.0230* | -0.0217* | -0.0211 |
|  | (0.0149) | (0.0132) | (0.0130) | (0.0128) | (0.0128) | (0.0128) | (0.0129) | (0.0128) |
| **Gender (reference: female)** |  |  |  |  |  |  |  |  |
| Male | -0.00873 | -0.00834 | -0.00876 | -0.00833 | -0.00901 | -0.00826 | -0.00853 | -0.00885 |
|  | (0.00953) | (0.00952) | (0.00952) | (0.00952) | (0.00953) | (0.00952) | (0.00952) | (0.00952) |
| **Education level (reference: medium)** |  |  |  |  |  |  |  |  |
| low education | 7.15e-05 | -0.000319 | 0.000613 | 0.00451 | 0.00115 | 0.00200 | 0.00120 | 0.000639 |
|  | (0.0242) | (0.0242) | (0.0242) | (0.0242) | (0.0242) | (0.0242) | (0.0242) | (0.0242) |
| high education | 0.0550*** | 0.0552*** | 0.0546*** | 0.0552*** | 0.0548*** | 0.0543*** | 0.0550*** | 0.0548*** |
|  | (0.00981) | (0.00980) | (0.00981) | (0.00980) | (0.00981) | (0.00980) | (0.00981) | (0.00981) |
| **Income level (reference: medium)** |  |  |  |  |  |  |  |  |
| low income | 0.0109 | 0.0105 | 0.0106 | 0.0105 | 0.0114 | 0.0102 | 0.0107 | 0.0111 |
|  | (0.0120) | (0.0120) | (0.0120) | (0.0120) | (0.0120) | (0.0120) | (0.0120) | (0.0120) |
| high income | -0.0445*** | -0.0447*** | -0.0447*** | -0.0444*** | -0.0445*** | -0.0450*** | -0.0448*** | -0.0443*** |
|  | (0.0114) | (0.0114) | (0.0114) | (0.0114) | (0.0114) | (0.0114) | (0.0114) | (0.0114) |
| **Risk attitude (reference: moderate)** |  |  |  |  |  |  |  |  |
| extremely risk loving | 0.194*** | 0.194*** | 0.195*** | 0.193*** | 0.194*** | 0.192*** | 0.194*** | 0.194*** |
|  | (0.0183) | (0.0183) | (0.0183) | (0.0183) | (0.0184) | (0.0183) | (0.0183) | (0.0183) |
| extremely risk averse |  |  |  |  |  |  |  |  |
|  | 0.0496*** | 0.0472*** | 0.0490*** | 0.0480*** | 0.0482*** | 0.0510*** | 0.0487*** | 0.0494*** |
|  | (0.00974) | (0.00952) | (0.00951) | (0.00964) | (0.00974) | (0.00958) | (0.00950) | (0.00949) |
|  |  |  |  |  |  |  |  |  |
| Year = 2020 | 0.0496*** | 0.0472*** | 0.0490*** | 0.0480*** | 0.0482*** | 0.0510*** | 0.0487*** | 0.0494*** |
|  | (0.00974) | (0.00952) | (0.00951) | (0.00964) | (0.00974) | (0.00958) | (0.00950) | (0.00949) |
| High regional and age risk | 0.0352 |  |  |  |  |  |  |  |
|  | (0.0308) |  |  |  |  |  |  |  |
| 2020 * High regional and age risk | -0.00665 |  |  |  |  |  |  |  |
|  | (0.0412) |  |  |  |  |  |  |  |
| Health shock and high age risk |  | -0.0693 |  |  |  |  |  |  |
|  |  | (0.0514) |  |  |  |  |  |  |
| 2020 * Health shock and high age risk |  | 0.198** |  |  |  |  |  |  |
|  |  | (0.0860) |  |  |  |  |  |  |
| Income shock and high age risk |  |  | -0.0964 |  |  |  |  |  |
|  |  |  | (0.125) |  |  |  |  |  |
| 2020 * Income shock and high age risk |  |  | 0.118 |  |  |  |  |  |
|  |  |  | (0.139) |  |  |  |  |  |
| Health shock and high regional risk |  |  |  | -0.0769** |  |  |  |  |
|  |  |  |  | (0.0360) |  |  |  |  |
| 2020 * Health shock and high regional risk |  |  |  | 0.0323 |  |  |  |  |
|  |  |  |  | (0.0509) |  |  |  |  |
| Income shock and high regional risk |  |  |  |  | -0.0389 |  |  |  |
|  |  |  |  |  | (0.0447) |  |  |  |
| 2020 * Income shock and high regional risk |  |  |  |  | 0.0432 |  |  |  |
|  |  |  |  |  | (0.0503) |  |  |  |
| Income shock and health shock |  |  |  |  |  | -0.0618 |  |  |
|  |  |  |  |  |  | (0.0531) |  |  |
| 2020 * Income shock and health shock |  |  |  |  |  | -0.0260 |  |  |
|  |  |  |  |  |  | (0.0648) |  |  |
| Health shock and high regional and age risk |  |  |  |  |  |  | -0.0962 |  |
|  |  |  |  |  |  |  | (0.0992) |  |
| 2020 * Health shock and high regional and age risk |  |  |  |  |  |  | 0.183 |  |
|  |  |  |  |  |  |  | (0.139) |  |
| Income shock and high regional and age risk |  |  |  |  |  |  |  | -0.292 |
|  |  |  |  |  |  |  |  | (0.278) |
| 2020 * Income shock and high regional and age risk |  |  |  |  |  |  |  | 0.272 |
|  |  |  |  |  |  |  |  | (0.300) |
| Constant | 0.416*** | 0.417*** | 0.416*** | 0.419*** | 0.417*** | 0.419*** | 0.417*** | 0.416*** |
|  | (0.0152) | (0.0152) | (0.0152) | (0.0152) | (0.0153) | (0.0153) | (0.0152) | (0.0152) |
|  |  |  |  |  |  |  |  |  |
| Observations | 3,569 | 3,569 | 3,569 | 3,569 | 3,569 | 3,569 | 3,569 | 3,569 |
| R-squared | 0.055 | 0.056 | 0.055 | 0.056 | 0.055 | 0.057 | 0.055 | 0.055 |

Note: Standard errors in parentheses. *** p<0.01, ** p<0.05, * p<0.1
